# High-order harmonic generation from a thin film crystal perturbed by a quasi-static terahertz field

**DOI:** 10.1038/s41467-023-38187-0

**Published:** 2023-05-05

**Authors:** Sha Li, Yaguo Tang, Lisa Ortmann, Bradford K. Talbert, Cosmin I. Blaga, Yu Hang Lai, Zhou Wang, Yang Cheng, Fengyuan Yang, Alexandra S. Landsman, Pierre Agostini, Louis F. DiMauro

**Affiliations:** Department of Physics, The Ohio State University, Columbus, OH 43210 USA

**Keywords:** Attosecond science, High-harmonic generation, Terahertz optics, Semiconductors

## Abstract

Studies of laser-driven strong field processes subjected to a (quasi-)static field have been mainly confined to theory. Here we provide an experimental realization by introducing a bichromatic approach for high harmonic generation (HHG) in a dielectric that combines an intense 70 femtosecond duration mid-infrared driving field with a weak 2 picosecond period terahertz (THz) dressing field. We address the physics underlying the THz field induced static symmetry breaking and its consequences on the efficient production/suppression of even-/odd-order harmonics, and demonstrate the ability to probe the HHG dynamics via the modulation of the harmonic distribution. Moreover, we report a delay-dependent even-order harmonic frequency shift that is proportional to the time derivative of the THz field. This suggests a limitation of the static symmetry breaking interpretation and implies that the resultant attosecond bursts are aperiodic, thus providing a frequency domain probe of attosecond transients while opening opportunities in precise attosecond pulse shaping.

## Abstract (web summary)

Here the authors establish a biochromatic method for ultrafast probe and control of the strong field high harmonic generation process in solid by combining strong mid-infrared and weak terahertz fields.

## Introduction

High harmonic generation (HHG) from centrosymmetric media driven by a monochromatic electromagnetic field forbids the production of even-order harmonics based on electric dipole transition within the Born-Oppenheimer approximation. There are several arguments why this is so. For one in phenomenological nonlinear optics, centrosymmetric media that possess inversion symmetry must have even-order nonlinear susceptibilities *χ*^2*n*^ ≡ 0, therefore ${P}_{2n\omega }\propto {\chi }^{2n}{F}_{\omega }^{2n}\equiv 0$^1^, here *P* is the polarization and *F* is the electric field. From the semiclassical perspective, even-order harmonic light generated from adjacent laser half-cycles interfere destructively and annihilate, due to a change in sign of the electron dipole, i.e., *d*(*t* + *T*/2) = − *d*(*t*). From a quantum viewpoint where the electromagnetic radiation is treated as photon, the absorption of even number of photons followed by the emission of one photon violates the conservation of parity and angular momentum and thus is forbidden. Strict proof based on Floquet group theory and electric dipole selection rules conclude that the emission of odd-order only harmonics results from the $\hat{{{{{{{{\bf{H}}}}}}}}}({{{{{{{\bf{r}}}}}}}},t)=\hat{{{{{{{{\bf{H}}}}}}}}}(-{{{{{{{\bf{r}}}}}}}},t+T/2)$ dynamical symmetry of the HHG Hamiltonian^2,3^.

**Fig. 1:**
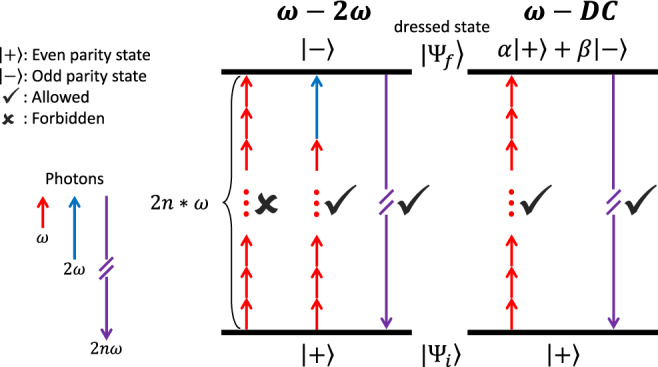
Energy diagrams for the *ω*-2*ω* and *ω*-DC driven even-order harmonic emission. The ground state of centrosymmetric media has a definite parity of either even or odd. For a weak 2*ω* field, the two dominant 2*nω* harmonic photon emission channels are those involving either the absorption or the emission (not shown in figure) of one 2*ω* photon.

Conversely, temporal and/or spatial breaking(s) of this Hamiltonian symmetry may promote even-order harmonic emission. For example, a phase-locked *ω*-2*ω* two-color field that is asymmetric in time generates even-order harmonics, and has proven to be a powerful time-resolved probe of the HHG process in gases^4,5^ and crystals^6^. In contrast, the application of a static (DC) electric field breaks the spatial symmetry of the medium and induces effective even-order susceptibilities *χ*^2*n*^, resulting in the production of even-order harmonics. For *χ*^2^ in solids, this is known as the electric field induced second harmonic generation (EFISHG)^7^, which has wide applications such as designing optical modulators^8^ and imaging electric fields^9^. It is worth noting that in quantum electrodynamics, the *ω*-2*ω* and *ω*-DC driven even-order harmonic emissions from centrosymmetric media are fundamentally different, as illustrated in Fig. 1. The former is essentially frequency mixing, with the total number of the absorbed *ω* and 2*ω* photons being odd to conserve the parity^10,11^. Whereas in the latter case, electrostatic fields cannot be quantized, they are conveniently called virtual photons and appear as intermediate interaction terms in Feynman diagrams. The absorption of even number of *ω* photons is allowed because in the presence of the static field, the dressed electron states have mixed parity.

**Fig. 2:**
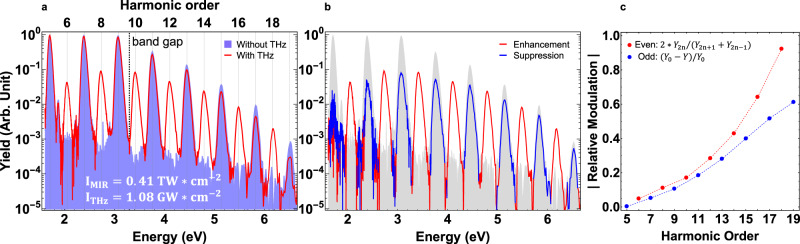
Modulation of the high harmonic distribution by the THz field. **a** Typical high-order harmonic spectra without (shaded blue) and with (red line) the THz field. **b** The difference spectrum from **a**, i.e., ∣*Y* − *Y*_0_∣, here *Y* and *Y*_0_ are harmonic yields with and without the THz field, respectively. Absolute values are taken to plot the difference spectrum on log-scale, red and blue colors are used to distinguish the enhancement (*Y* > *Y*_0_) and suppression (*Y* < *Y*_0_) of the yield. For reference, the shaded gray is the spectrum without the THz field. **c** Blue: the relative suppression of odd-order harmonic, 1 − *Y*/*Y*_0_, red: the even-order harmonic yield relative to adjacent odd-orders, 2*Y_2*n*_/(*Y*_2*n*−1_ + *Y*_2*n*+1_), as a function of the harmonic order, at *I*_MIR_ = 0.41 TW cm^−2^ and *I*_THz_ = 1.08 GW cm^−2^.

**Fig. 3:**
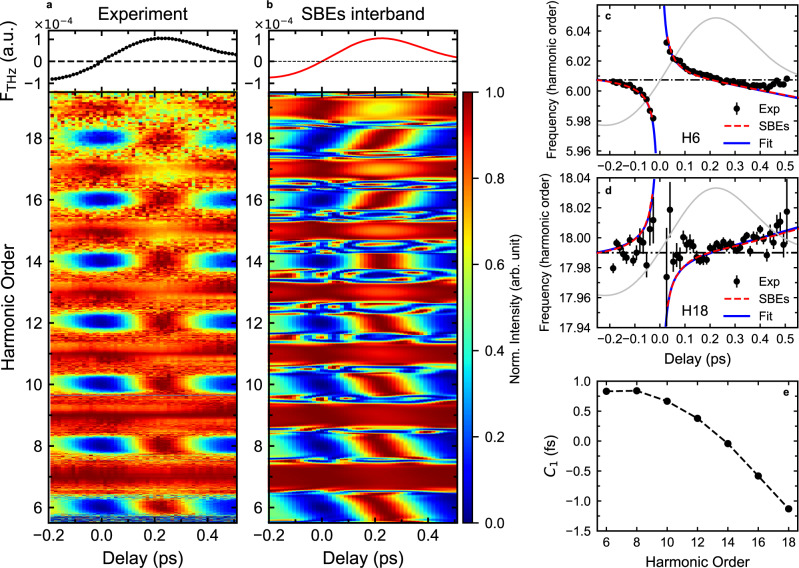
Delay-dependent even-order harmonic frequency shift. “Normalized" HHG spectrogram as a function of the MIR-THz field delay, at *I*_MIR_ = 0.74 TW cm^−2^ and *I*_THz_ = 1.08 GW cm^−2^: Experiment (**a**) and SBEs interband simulation (**b**). For a clearest visualization of the very small even-order harmonic frequency shifts, we perform the following normalization: in the figures, each energy pixel “row" is individually normalized to their maximum. The width of the row corresponds to the energy resolution of the harmonic spectrum. In the experiment, each row (pixel) corresponds to a wavelength width of ~ 0.56 nm. In the simulation, each row corresponds to an energy width of ~ 1.4 meV (0.004*ω*_MIR_). Centro-frequencies of the 6th (**c**) and the 18th (**d**) order harmonics as a function of the MIR-THz field delay: Experiment, extracted from Gaussian fit of the harmonic peak (black dots with error bars), SBEs interband (red dashed curve), Fitting to SBEs interband with the equation ${\omega }_{2n}/{\omega }_{0}={C}_{0}+{C}_{1}*{F}_{{{{{{{{\rm{THz}}}}}}}}}^{{\prime} }(t)/{F}_{{{{{{{{\rm{THz}}}}}}}}}(t)$ (blue solid curve), SBEs interband with a 540 kV cm^−1^ static field (black dash-dotted line). The gray curve shows the THz waveform, delay zero is at the time when the peak of the MIR pulse overlaps with the zero-crossing of the THz field. **e** The fitting parameter *C*_1_ as a function of the harmonic order.

In this work, we introduce a variant two-color scheme that is intermediate to the two cases discussed above. A strong MIR driving field is combined with a weak THz dressing field, to generate high-order harmonics from a ZnO thin film crystal. The THz field can be considered quasi-static since its period (~2 ps) is much longer than the MIR pulse duration (~70 fs). A distinct feature of the THz field is that its ponderomotive potential far exceeds that of the MIR field, which is not the case in previous studies with *ω*-2*ω* drive. Thus, in our study the perturbation of the HHG process occurs in the Keldysh low-frequency regime, approaching the static limit. Figures 2 and 3 summarize our main experimental observations. In the presence of the THz field, we observe not only efficient generation of very high even-order harmonics (~5% for the 6th-order, ~90% for the 18th-order), but also commensurate suppression of the odd-orders. The relative generation/suppression of the even-/odd-orders increase monotonically as a function of the harmonic order. In addition, we observe a delay dependent frequency shift of the even-order harmonics. One caveat, only in the limiting case of a static perturbing field are strict even-/odd-order harmonics allowed from the MIR drive, instead the presence of the quasi-static THz field causes the MIR field to lose its periodicity (*T*_0_ ≡ 2*π*/*ω*_MIR_), i.e., *F*(*t*) ≠ *F*(*t* + *T*_0_) for the total field. For HHG in the time domain, this equates to phase-locked aperiodic attosecond bursts, and in the frequency domain leads to emission of light that are anharmonics of the fundamental. However, for simplicity, we will continue to refer to even-/odd-orders in the manuscript. In the experiment, the effect of the THz field being non-static manifests itself as very small, yet measurable, shifts of the even-order harmonic frequencies as the delay between the MIR and THz fields is varied.

To the best of our knowledge, the high even-order harmonic conversion efficiency, the suppression of the odd-order harmonics, the monotonic order-dependent modulation of the harmonic yield, and the delay-dependent frequency shift of the even-order harmonics, have never been reported in a dielectric with a quasi-static perturbing field. A recent report aimed at utilizing HHG for field imaging demonstrated the ability to image electric field in the vicinity of nano-electrodes using photonic enhancement of even-order harmonic light induced by a weak static or THz field^12^. The observed conversion efficiencies in nano-patterned ZnO and Si crystals were extremely weak (~0.1%) and only for low even-orders (≤8th-order). In contrast, our study aims to probe the basic strong field processes of harmonic generation in dielectrics subjected to a quasi-static field using high dynamic range experimental measurements and comprehensive theoretical modeling. We exploit our experimental observables as robust time-resolved probes of the HHG process. Analogous to the semiclassical three-step model^13,14^ that describes the electron motion in the gas-phase, interband HHG in solids can be interpreted as a generalized recollision process^6,15^: An electron–hole pair is created (at the minimum band gap, i.e., Γ-point) via excitation of the electron from valence to conduction band, the electron and hole are then accelerated and separated by the laser electric field, and as the field changes sign, they are driven back to each other to recombine, emitting photons with energy that equals the band gap at recombination. We find that in the presence of the THz perturbing field, the electron/hole accumulates an extra dipole phase difference (*δϕ*) during propagation, which is closely related to the classical action (*S*) and the recombination time (*t*_c_). The order-dependent modulations of the harmonic yield and/or frequency indicate that *S*, *t*_c_, and *δϕ* depend on the harmonic order, thus conveying information about the electron dynamics, the quantum trajectories and the band structure.

## Results

In the experiment, a strong 70 fs, 3.6 μm (83.3 THz, 0.34 eV) MIR pulse is combined with a phased-locked weak ~2 ps, 430 μm (0.7 THz, 2.9 meV) asymmetric single-cycle THz pulse. The two-color field drives HHG from a 100 nm thickness, c-plane (0001) poly-crystalline ZnO thin film. Unless otherwise specified, the MIR pulse temporally overlaps with the crest of the THz pulse. We focus on the configuration where the MIR and THz fields are linearly polarized and parallel. Some results with orthogonal MIR-THz drive are presented in Supplementary note 1.

### Harmonic yield scaling with the MIR and THz intensities

**Fig. 4:**
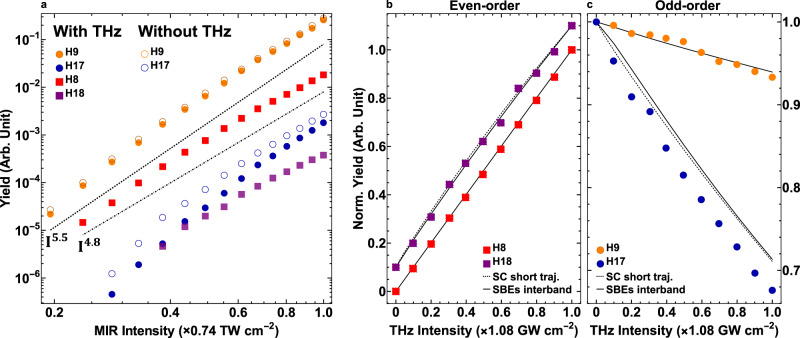
Harmonic scaling with respect to the MIR and THz intensities. **a** Harmonic yield as a function of the MIR intensity at a fixed *I*_THz_ = 1.08 GW cm^−2^. Dashed and dash-dotted lines are power scale guidelines. Even-order (**b**) and odd-order (**c**) harmonic yield as a function of the THz intensity at a fixed *I*_MIR_ = 0.74 TW cm^−2^. Each harmonic order is individually normalized to their maximum, and the 18th-order is y-offset by 0.1 for clearer visualization. Solid and dashed lines are the SBEs interband simulation and the semiclassical short trajectory calculation, respectively. See Supplementary Fig. 5 for the full experimental results of the harmonic scaling from the 5th to the 19th orders.

In order to confirm the excitation regime, we show in Fig. 4a that the measured harmonic yield, both odd and even, as a function of the MIR intensity *I*_*ω*_, is roughly independent of the harmonic order *q*, with a power law scaling of approximately ${I}_{\omega }^{5}$, indicative of a non-perturbative excitation. This is at variance with the ${I}_{\omega }^{q}$ multiphoton perturbative scaling and the linear scaling reported in ref. ^12^. In addition, we show in Supplementary Fig. 1 that the even-order harmonic conversion ratio between the parallel and perpendicular MIR-THz polarization configurations depends on the harmonic order and varies from ~50:1 to ~6:1, also deviates from the perturbative expectation of ~10:1^16^. On the other hand, Fig. 4b, c shows that the yield of each even-/odd-order harmonic increases/decreases linearly with the THz intensity, suggesting that the THz field is a first-order perturbation to the HHG process.

### THz field-induced modulation of the harmonic distribution

Typical HHG spectra, without and with the THz field applied, are shown in Fig. 2a, from which we can extract the order-dependent modulation of the harmonic yield. The relative suppression of the odd-order harmonic, 1 − *Y*/*Y*_0_, where *Y* and *Y*_0_ are respectively the yields with and without the THz field, and the even-order harmonic yield relative to its adjacent odd-orders, 2*Y_2*n*_/(*Y*_2*n*−1_ + *Y*_2*n*+1_), are plotted in Fig. 2c. Both ratios increase monotonically with increasing harmonic order and remarkably, the highest orders experience extremely large modulations, for example, the 18th and 19th orders approach ~90% and ~60% relative generation/suppression, respectively. In comparison, the *ω*-2*ω* two-color field in ref. ^6^ showed no regularity in the even-order relative scaling nor clear odd-order modulation in the weak (perturbative) second harmonic field. The different behavior with the current study is indicative of the different Keldysh scaling limit of the THz perturbing field. This is further supported in Supplementary Note 1, by comparing the order-dependence of the relative even-order harmonic conversion from orthogonal MIR-THz drive with previous study from orthogonal *ω*-2*ω* drive^17^. Higher-/lower-orders are more efficient in the former/latter case, respectively.

### Delay-dependent even-order harmonic frequency shift

In *ω*-2*ω* driven HHG, attosecond electron dynamics is extracted by recording the HHG spectrogram as a function of the relative delay between the two colors^4–6^. In contrast our MIR-THz field makes resolving such harmonic phases challenging, due to the large difference in time scale between the MIR and THz periods. However, in our experiment, we access a new observable that depends on the MIR-THz field delay (Fig. 3): an order-dependent frequency shift of even-order harmonics that changes not only in magnitude but also in sign. As will be discussed, the delay-dependent frequency shift follows an expression that simply depends on the THz waveform and the harmonic order, this is illustrated in Fig. 3c, d for the 6th and 18th orders, respectively.

## Discussion

**Fig. 5:**
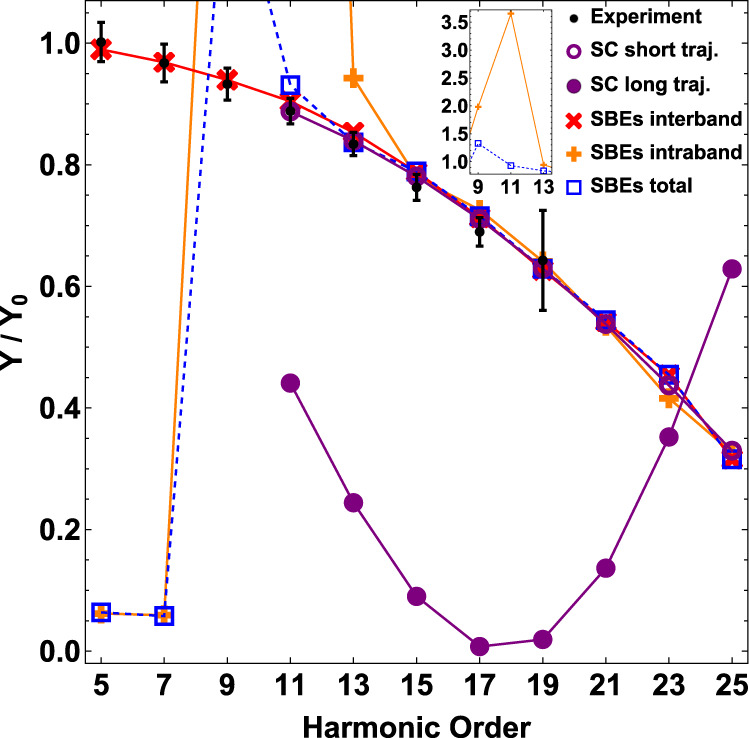
Comparison of experiment with the semiclassical (SC) calculation and the SBEs simulation for the order-dependent modulation of the odd-order harmonic yield. The MIR and THz intensities are *I*_MIR _= 0.74 TW cm^−2^ and *I*_THz_ = 1.08 GW cm^−2^, respectively. *Y* and *Y*_0_ are harmonic yields with and without the THz field, respectively. Error bars are standard deviations of 10 measurements. See Supplementary Fig. 3 for experimental harmonic yield modulation at various MIR intensities.

In essence, our experimental results reveal a dichotomy in the quasi-static interpretation of the THz field. To provide further clarity into the THz dressed HHG process, we compare the experiment with simulations based on the semiconductor Bloch Equations (SBEs)^15^. A description of the theoretical method is provided in the METHOD section while detailed results are presented in Supplementary Note 4. Comparisons between the experimental results and the SBEs simulations verify that above-band gap harmonics are dominated by the interband polarization^6,15^ but surprisingly, this also applies to below-band gap harmonics. Conversely, the intraband current which originates from (dynamical) Bloch oscillations of the electron/hole within a single band^18–20^, fails in reproducing our observable of the frequency shift (Supplementary Fig. 11), and the generation/suppression of even-/odd-order harmonics that depend linearly on the THz intensity (Supplementary Fig. 9), as well as their order-dependence (Fig. 5). Hence, hereafter we will limit our discussion to only the interband process and summarize below the main physical insights from the simulation.

The linear dependence of the harmonic yield on the THz intensity is accurately reproduced (Supplementary Fig. 9). Moreover, we find that as the delay between the MIR and THz fields is varied, both the even-/odd-order harmonic generation/suppression are in phase with the temporal profile of the THz intensity (Supplementary Fig. 10), validating the treatment of the THz induced symmetry breaking being static: the harmonic emission is impacted only by the instantaneous THz field during the sub-cycle MIR driven HHG time while the THz carrier frequency plays little role. On the other hand, a frequency mixing process that sums odd numbers of MIR and THz photons is also possible, but only under the condition that the MIR pulse duration covers at least one cycle of a multi-cycle THz pulse, i.e., the breakdown of the quasi-static approximation.

Intuitively, it may be interpreted that the spatial symmetry of the crystal is reduced and even-order harmonic emission is promoted via the THz field induced (static) effective even-order susceptibilities ${\chi }_{{{{{{{{\rm{eff.}}}}}}}}}^{2n}={\chi }^{2n+1}{F}_{{{{{{{{\rm{THz}}}}}}}}}$^16^. However, for non-perturbative HHG, like our experiment, calculating the *q*^th^ harmonic based on ${P}_{q\omega }\propto {\chi }^{q}{F}_{\omega }^{q}$ where *χ*^*q*^ is time-independent is no longer valid, and is inconsistent with the suppression of odd-order harmonics. Therefore we examine the THz perturbation to the HHG process using a semiclassical approach.

**Fig. 6:**
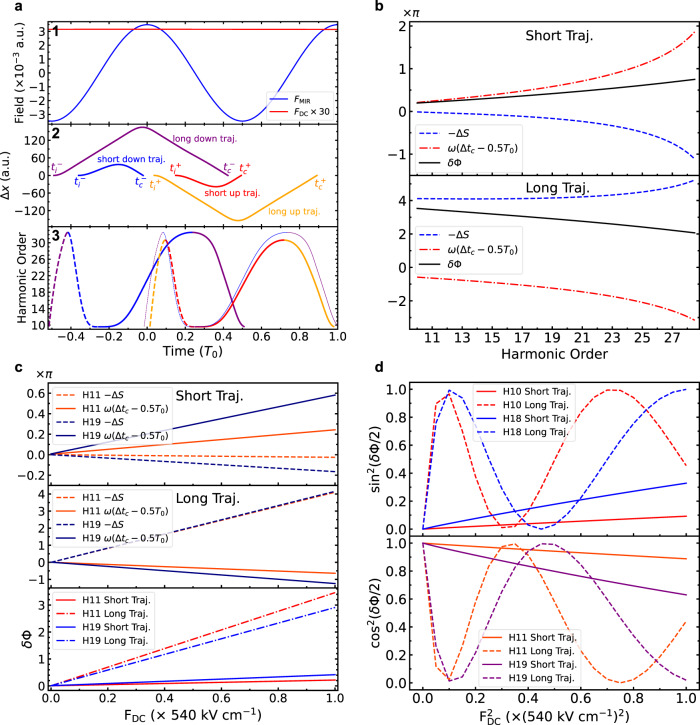
A typical semiclassical analysis of the HHG process perturbed by a static field. **a1** Temporal profiles of the *cw*-MIR field and the static field used in the calculations. **a2** For four electron trajectories associated with the 19^th^-order harmonic, separation of the electron-hole pair, Δ*x*, as a function of time. *t*_i_ and *t*_c_ are the excitation and recombination times, respectively. **a3** Harmonic photon energy as a function of the excitation (thick dashed curve) and recombination (thick solid curve) times: short down trajectory (blue), long down trajectory (purple), short up trajectory (red), long up trajectory (orange). The thin curve is a copy of the down trajectories shifted in time by 0.5*T*_0_, which should overlap with the up trajectories in the absence of the static field. **b** The extra dipole phase difference, *δϕ* = *ω*(Δ*t*_*c*_ − 0.5*T*_0_) − Δ*S*, as a function of the harmonic order at *F*_DC_ = 540 KV cm^−1^. For selected harmonic orders, *δϕ* as a function of *F*_DC_ (**c**), and the relative even-/odd-order harmonic yields as a function of ${F}_{{{{{{{{\rm{DC}}}}}}}}}^{2}$ (**d**). I_MIR_ = 0.74 TW cm^−2^ has been used in all these calculations.

Based on the saddle point method, the integral that is central to the interband transition amplitude in the SBEs has its dominant contribution from the stationary phase points^15^, which correspond to the semiclassical electron trajectories during the generalized recollision process. This suggests that the electron dynamics can be described by a semiclassical treatment of the electron motion driven by the MIR field in the presence of the THz field, accessing the electron-hole recombination time *t*_c_ and the classical action *S* for each above-band gap harmonic. A detailed description of the semiclassical analysis is provided in the METHOD section while Fig. 6 is the result of a typical calculation. We demonstrate that the THz field modulates the high harmonic distribution via the introduction of a first-order perturbation to the electron dipole phase difference between adjacent MIR field half-cycles, *δϕ* = *ω*(Δ*t*_c_ − 0.5*T*_0_) − Δ*S* ∝ *F*_THz_, and the even-/odd-order harmonic yields are respectively proportional to ${\sin }^{2}(\delta \phi /2)$ and ${\cos }^{2}(\delta \phi /2)$, here *ω* is the harmonic frequency. Importantly, in contrast to refs. ^4,12^, in which only the change in the classical action (Δ*S*) is considered, we identify that including the modification of the electron-hole recombination time (*t*_c_) is critical for a quasi-static perturbing field.

In the semiclassical picture, each harmonic order arises from two distinct electron trajectories with different birth times in a MIR half-cycle and repetitive at *ω*_MIR_, they are identified as the short and long trajectories based on their different excursion times between excitation and recombination. Our analysis reveals that the THz dressed HHG process can distinguish between these two trajectories. We find that only calculations of the short trajectories reproduce both the linear dependence of the harmonic yield on the THz intensity (Figs. 4, 6) and the monotonic increasing relative odd-order suppression (Fig. 5). In fact, none of the measurements are consistent with a long trajectory interpretation, perhaps indicative of the rapid electron-hole decoherence. In contrast to spectrogram measurements of phases^4^ or coherence times^21^, the THz method provides an alternate, single-shot means for discriminating the short and long trajectories. In the future, experiments using shorter wavelength drives and/or cryogenic cooling of the crystal could make harmonics generated from the long trajectories, if existing, observable. In addition to the probe of quantum trajectories, comparison of heavy holes, light holes, and split-off valance bands calculations suggests that it is also possible to probe the crystal band structure via the harmonic yield modulation. A brief discussion is included in Supplementary Note 2.

The observed MIR-THz delay-dependent frequency shift of even-order harmonics cannot be explained by a static perturbing field. We find in our SBEs simulation that replacing the THz field with a static field (${F}^{{\prime} }(t)\equiv 0$) results in harmonic frequencies that are independent of the static field strength, suggesting that the THz field derivative is playing a central role in the emitted harmonic frequencies. We closely investigate the key factor(s) that determine the frequency shift by performing a series of SBEs simulations for different MIR and THz parameters. The results are summarized in Supplementary Fig. 13, which shows that the frequency shift is not sensitive to the (multi-cycle) MIR pulse duration, temporal pulse shape, carrier-envelope-phase (CEP), the MIR and THz intensities, and the dephasing time applied in the Bloch equations. In addition, we provide SBEs simulations in Supplementary Note 5 showing that the frequency shift is dependent on the MIR wavelength, group delay dispersion (GDD), (few-cycle) short pulse CEP, and the crystal band gap. Surprisingly, we find that a simple equation fits the delay-dependent even-order harmonic frequency very well and indicates that it is proportional to the THz field derivative divided by the THz field, i.e., ${\omega }_{2n}/{\omega }_{0}={C}_{0}+{C}_{1}*{F}_{{{{{{{{\rm{THz}}}}}}}}}^{{\prime} }(t)/{F}_{{{{{{{{\rm{THz}}}}}}}}}(t)$, and the fitting parameters *C*_0_ and *C*_1_ depend on the harmonic order. The small offset *C*_0_ − 2*n* ≠ 0 originates from the MIR field being pulsed, and is dependent on the MIR pulse duration. The *C*_1_ parameter describes the amplitude and sign of the frequency shift. The term ${F}_{{{{{{{{\rm{THz}}}}}}}}}^{{\prime} }(t)/{F}_{{{{{{{{\rm{THz}}}}}}}}}(t)$ implies that the frequency is determined by the THz temporal waveform. The order-dependence of the frequency shift is reflective of the different electron excursion times for different harmonic orders. In the presence of the THz perturbing field, the extra energy the electrons gain/lose, which changes from cycle-to-cycle of the MIR field-driven recollision (i.e., temporally aperiodic), depends on both how fast $\left({F}^{{\prime} }(t)\right)$ and how strong $\left(F(t)\right)$ the THz field is. This quasi-static effect is imprinted onto the frequencies of the emitted harmonic light. Therefore, the THz field alters in a well-defined way the high harmonic frequencies, opening up opportunities in attosecond pulse shaping, and providing a probe of attosecond transients in the frequency domain.

In the end, we propose two potential applications of the THz-dressed HHG. The linear dependence of the harmonic yield on the instantaneous THz intensity can be utilized for precise THz metrology, as we demonstrate in Supplementary Note 3. This THz waveform sampling technique is not bandwidth-limited by electro-optical properties of the crystal, thus has the potential for developing ultra-broadband, ultra-sensitive THz time domain spectroscopy (THz-TDS). Even-order harmonic emission induced by electric field naturally provides a method for imaging the field distribution. In ref. ^12^, dynamical imaging of electric field near metallic nano-structures by the 4th-order harmonic light at 500 nm has been demonstrated. Using high-order harmonics in the extreme-ultraviolet regime driven by ultrafast laser pulses could in principle realize nanometer/femtosecond spatial/temporal resolutions.

In conclusion, we successfully demonstrate a method to study the strong field HHG process in solid utilizing the MIR-THz two-color field, paving the way for ultrafast probe and control of HHG by THz field. Future studies that vary the polarization and/or ellipticity of the MIR and/or THz fields, and the orientation of single-crystals, could further unravel the fundamentals of HHG in solids. Perturbing and/or controlling HHG in gases by (quasi-)static electric field have been proposed in the past but only theoretical investigations exist^22–27^. Our study builds an alternative platform to test these theories in solids, and as table-top THz sources approach field strengths of tens of MV cm^−1^ ^28^, provides guidance for future gas-phase, THz-dressed HHG experiments.

## Methods

### Experimental setup

In the experiment, 70 fs (intensity FWHM), 3.6 μm (83.3 THz, 0.34 eV) MIR pulses are generated from an optical parametric amplifier based on KTA crystals. ~2 ps, 430 μm (0.7 THz, 2.9 meV) asymmetric single-cycle THz pulses are generated via tilted-pulse-front-pumping (TPFP) optical rectification in a LiNbO_3_ crystal. Both the MIR and the THz generations are pumped by laser pulses delivered from the same Ti:sapphire system (80 fs, 800 nm, 5.5 mJ, 1 kHz). Group delay dispersion (GDD) of the MIR light from transmissive optics (lens, waveplates, polarizers, substrates etc.) has been carefully compensated to obtain the shortest pulse. FROG measurement indicates that a transform limited pulse has a duration of around 60 fs. Depending on detailed characteristics of the 800 nm pump pulse and fine alignment of the pump beam, the THz pulse shape (asymmetry between positive and negative half-cycles, small satellite cycles before/after the main cycle) and centro-frequency may have small day-to-day variations. However, these do not affect our study in this manuscript. The sample used in the experiment is a 100 nm thickness, c-plane polycrystalline ZnO thin film (band gap ~3.3 eV) deposited on 0.5 mm thickness fused silica substrate (MTI Corporation). Even-order harmonic emission is NOT observed from the sample with the MIR drive alone at normal incidence. And at the MIR intensities used in the experiment, the harmonic generation is isotropic, i.e., the harmonic yield does not depend on the sample orientation with respect to the MIR polarization.

The MIR and THz pulses incident normal to and are focused at the ZnO thin film. The sample is mounted backwards (the lasers hit the substrate first) to avoid absorption of harmonic light by the substrate. We do not observe significant distortions of the MIR pulse by the substrate. The focal size (1/*e*^2^ intensity diameter) of the MIR and THz beams are ~240 μm and ~1.5 mm, respectively. Therefore, the THz intensity can be considered uniform within the MIR focus. The MIR intensity (in air) is calibrated geometrically via measurements of pulse duration, pulse energy, and focal size. The THz intensity (in air) is estimated from electro-optic sampling. Accounting for the reflection losses and constructive thin film interferences, the maximum applied MIR and THz peak intensities (fields) in the ZnO film are 0.74 TW cm^−2^ (18 MV cm^−1^) and 1.08 GW cm^−2^ (540 KV cm^−1^), respectively. Harmonic light is collected by a visible-vacuum ultraviolet (VIS-VUV) monochromator (120–900 nm) and detected by a cooled ICCD camera (16 bits, noise level < 5). In the experiment, we observe high-order harmonics up to the 19th order, or 6.55 eV (189 nm) in photon energy (wavelength), the detection of higher energy photons is limited by the experiment being performed in ambient air.

The standard deviation of the 3.6 μm MIR intensity is about 0.2–0.4%, and that of the high-order harmonic yields typically stay around 1–3%. To extract the relative suppression of the odd-order harmonics, and the very small change (1–2%) in the total harmonic yield with good statistics, we perform back-to-back measurements of the harmonic spectrum without and with the THz field, and repeat the measurements many times. For these harmonic yield modulation measurements, more than 10^6^ laser shots are averaged to obtain a single spectrum.

### Semiconductor Bloch model

The semiconductor Bloch model describes the coupled interband and intraband dynamics for HHG in solids. We solve the following one-dimensional, two-band semiconductor Bloch equations (SBEs)^15,29^,

1 $${\dot{n}}_{m}(K,\,t)=i{s}_{m}F(t)\cdot d^*\left(K+A(t)\right)\pi (K,\,t)+c.c.$$

2 $$\dot{\pi }(K,\,t)=\left[i{\epsilon }_{g}\left(K+A(t)\right)-\frac{1}{{T}_{2}}\right]\pi (K,\,t)-iF(t)\cdot d\left(K+A(t)\right)w(K,\,t)$$

here the crystal momentum *k* has been transformed into a moving frame, *k* → *K* = *k* − *A*(*t*). The interband polarization *p*(*K*, *t*) is related to *π*(*K*, *t*) by *p*(*K*, *t*) = *d*(*k*)π^*^(*K*, *t*) + *c*.*c*. A variable transformation *π* → *πe*^*iS*(*K*, *t*)^, with $S(K,\,t)=\int\nolimits_{-\infty }^{t}{{{{{{{\rm{d}}}}}}}}\tau \,{\epsilon }_{g}\left(K+A(\tau )\right)$ the classical action, has been performed for computational convenience^29^. *n*_*m*_ are the valence (*m* = *v*) and conduction (*m* = *c*) band populations, and their difference *w* = *n*_*v*_ − *n*_*c*_ is set as 1 for low ionization limit. *s*_*m*_ = − 1, 1 for *m* = *v*, *c*, respectively. *ϵ*_*g*_(*k*) = *E*_*c*_(*k*) − *E*_*v*_(*k*) is the band gap, and the band dispersion of ZnO is taken from ref. ^30^, with a band gap of 3.3 eV at the Γ point. $d(k)=ie\cdot \int\,{{{{{{{\rm{d}}}}}}}}{{{{{{{\bf{x}}}}}}}}\,{u}_{v,k}^{*}({{{{{{{\bf{x}}}}}}}}){\nabla }_{k}{u}_{c,k}({{{{{{{\bf{x}}}}}}}})$ is the transition dipole moment, with *e* the electron charge and *u*_*m*,*k*_ the periodic part of the Bloch function. In the calculation, *d*(*k*) is set to be 3.46, neglecting the *k* dependence^15^. The dephasing time *T*_2_ that describes the de-coherence of the electron-hole pair is chosen to be 1.0 fs. *F*(*t*) is the sum of the MIR and THz fields. The MIR pulse has a Gaussian profile, ${F}_{{{{{{{{\rm{MIR}}}}}}}}}(t)={F}_{{{{{{{{\rm{MIR}}}}}}}}}*{e}^{-2\ln 2{(t/{\tau }_{{{{{{{{\rm{MIR}}}}}}}}})}^{2}}\cos ({\omega }_{{{{{{{{\rm{MIR}}}}}}}}}t)$, with a pulse duration *τ*_MIR_ = 70 fs. The single-cycle THz pulse (*f* ≃ 0.7 THz) has a vector potential, ${A}_{{{{{{{{\rm{THz}}}}}}}}}(t)=-\frac{{F}_{{{{{{{{\rm{THz}}}}}}}}}{t}_{w}}{{C}_{0}}{e}^{-[1+\frac{1}{a}\tanh (bt/{t}_{w})]{(t/{t}_{w})}^{2}}$^31^. *a* = 1.226, *b* = 0.3, *C*_0_ = 0.988 and *t*_*w*_ = 0.325 ps are chosen to match with the measured THz waveform. The polarization of the MIR and THz fields is set along the Γ-*M* direction. However, simulations with the fields polarized along the Γ-*K* direction yield no significant differences up to the 25^th^-order harmonic, in agreement with the near-isotropic ZnO band structure below ~8 eV. The simulation is performed in length gauge over the entire THz pulse duration, i.e., from −1.8 to +1.2 ps (see Supplementary Fig. 7 for the pulse shapes).

After numerically solving the SBEs, the intraband *j*_intra_ and interband *j*_inter_ contributions to the total current *j* = *j*_intra_ + *j*_inter_ are obtained by,

3 $${j}_{{{{{{{{\rm{intra}}}}}}}}}(t)=\mathop{\sum}\limits_{m=c,v}{\int}_{\overline{{{{{{{{\rm{BZ}}}}}}}}}}{{{{{{{\rm{d}}}}}}}}K\,e{v}_{m}\left(K+A(t)\right)\cdot {n}_{m}(K,\,t)$$

4 $${j}_{{{{{{{{\rm{inter}}}}}}}}}(t)=\frac{{{{{{{{\rm{d}}}}}}}}}{{{{{{{{\rm{d}}}}}}}}t}{\int}_{\overline{{{{{{{{\rm{BZ}}}}}}}}}}{{{{{{{\rm{d}}}}}}}}K\,p(K,\,t)$$

here *v*_*m*_(*k*) = ∇_*k*_*E*_*m*_(*k*) is the group velocity. The harmonic spectra can then be obtained from the Fourier transform (FT) of the total current, Yield ∝ ∣FT{*j*(*t*)}∣^2^.

Supplementary Fig. 7 shows typical harmonic spectra from the simulation. From the spectra alone, for below-band gap harmonics, the intraband contribution far exceeds the interband, and the yields of even-orders (the 6th and 8th) are comparable to adjacent odd-orders, whereas in the experiment, they are relatively a few percent of the adjacent odd-orders. More importantly, the behaviors of harmonics generated from the intraband transition disagree with all of the experiment observations (Fig. 5, Supplementary Figs. 9–11). This provides a new perspective. The generalized recollision interpretation of the interband transition suggests that it only produces above-band gap harmonics, but apparently, our experiment supports that the below-band gap harmonics are also dominated by the interband transition, in agreement with previous studies^6,32^. Meanwhile, we think some correction to the SBEs is necessary in order to more accurately calculate the intraband current, especially for the below-band gap harmonics. For example, the ultrafast dephasing (*T*_2_, on the order of 1–2 fs) only affects the interband dynamics, but damping of the intraband electron current (typically on sub-10 fs time scale) is not considered in the SBEs. It is known that due to ultrafast scattering which can easily destroy the coherence of the Bloch states, (intraband) Bloch oscillation is typically realized in artificial structures (for example, superlattice) and at very low temperatures. For HHG in a realistic system, excluding the intraband current damping may result in an overestimation of the intraband contribution to the harmonic emission. A detailed analysis of this problem is beyond the scope of this work. In ref. ^20^, intraband current damping is introduced in the SBEs for HHG from GaSe driven by a 9 μm long-wavelength IR pulse.

### Semiclassical analysis of the above-band gap harmonics

In the following, we treat HHG in solids semiclassically by considering the generalized recollision process, which corresponds to the (above-band gap) interband transition of the semiconductor Bloch model. Under weak field approximation, the THz influence(s) to the excitation process can be neglected. We have confirmed in the SBEs simulation that the THz induced imbalance in the photocarrier excitation probabilities between adjacent MIR half-cycles only introduces a ~4% amplitude difference of the corresponding interband currents (Supplementary Fig. 14). As will be shown, this only results in a ~0.16% relative harmonic yield modulation, hence can be ignored for the above-band gap harmonics. In addition, the THz field induced Franz-Keldysh effect does not alter the MIR transmission/absorption, as the MIR photon energy is far below the ZnO band gap. The weak THz field does not affect the HHG transition dipole matrix elements and the amplitude of the transition dipole remains unaltered, the perturbation mainly acts on the dipole phase the electron/hole accumulates during propagation in “continuum”.

We first analyze without considering the THz induced imbalance in the photocarrier excitation. In low ionization limit, the interband current in the frequency domain can be expressed as,

5 $${j}_{{{{{{{{\rm{inter}}}}}}}}}(\omega )=-i\omega \int\nolimits_{-\infty }^{\infty }{{{{{{{\rm{d}}}}}}}}t\,{e}^{i\omega t}\left[{\int}_{{{{{{{{\rm{BZ}}}}}}}}}{{{{{{{\rm{d}}}}}}}}k\,d(k)\int\nolimits_{-\infty }^{t}{{{{{{{\rm{d}}}}}}}}{t}^{{\prime} }\,iF({t}^{{\prime} })\cdot {d}^{*}({k}_{{t}^{{\prime} }}){e}^{-iS(k,{t}^{{\prime} },t)}{e}^{-(t-{t}^{{\prime} })/{T}_{2}}+c.c.\right]$$

here ${k}_{{t}^{{\prime} }}=k-A(t)+A({t}^{{\prime} })$ and $S(k,\,{t}^{{\prime} },\,t)=\int\nolimits_{{t}^{{\prime} }}^{t}d\tau {\epsilon }_{g}({k}_{\tau })$. Based on the saddle point method, the dominant contribution to the integral comes from places where the first derivative of the phase, $\phi=\omega t-S(k,\,{t}^{{\prime} },\,t)$, is zero. These stationary phase points $\{k,\,{t}^{{\prime} },\,t\}$ satisfy the equations *k*(*τ*) = *A*(*τ*) − *A*(*t*_i_), ${{\Delta }}x=\int\nolimits_{{t}^{{\prime} }={t}_{{{{{{{{\rm{i}}}}}}}}}}^{t={t}_{{{{{{{{\rm{c}}}}}}}}}}{{{{{{{\rm{d}}}}}}}}\tau \,[{v}_{c}\left(k(\tau )\right)-{v}_{v}\left(k(\tau )\right)]=0$, and $\omega={\epsilon }_{g}\left(k({t}_{{{{{{{{\rm{c}}}}}}}}})\right)$. They correspond to the laser electric field driven semi-classical trajectories of the electron/hole between the excitation *t*_i_ and the recombination *t*_c_. The harmonic emissions occur at times *t*_c_ and the emitted photon energies equal the band gap at *t*_c_. The integral can then be estimated as sum of the integrand at all the saddle points,

6 $${j}_{{{{{{{{\rm{inter}}}}}}}}}(\omega )\approx \mathop{\sum}\limits_{{t}_{{{{{{{{\rm{c}}}}}}}}}}\omega F({t}_{{{{{{{{\rm{i}}}}}}}}})g({t}_{{{{{{{{\rm{c}}}}}}}}}){e}^{-({t}_{{{{{{{{\rm{c}}}}}}}}}-{t}_{{{{{{{{\rm{i}}}}}}}}})/{T}_{2}}{e}^{i\omega {t}_{{{{{{{{\rm{c}}}}}}}}}-iS({t}_{{{{{{{{\rm{i}}}}}}}}},{t}_{{{{{{{{\rm{c}}}}}}}}})}$$

here *g*(*t*_c_) accounts for the effective integration volume and the transition dipole moment. Multiple recollisions are neglected due to ultrafast dephasing, therefore the excitation and recombination times *t*_i_ and *t*_c_ are one to one correspondent.

To simplify the calculation, the MIR field is treated as a continuous wave (*cw*) like N-cycle pulse, and the THz field is replaced by a (positive) static field. For calculations of the harmonic yield, these are reasonable approximations for the multi-cycle MIR pulse and the quasi-static THz pulse in our experiment. We separate the electron trajectories into two, defined as the “up” (+) and “down” (−) trajectories with the MIR field being positive or negative at the time of excitation *t*_i_, respectively. A static perturbing field does not break the time-periodicity of the MIR field and the HHG process repeats every MIR field cycle. Therefore the interband currents for the up and down trajectories can be written as,

7 $${j}_{{{{{{{{\rm{inter}}}}}}}}}^{+}(\omega )=\omega F({t}_{{{{{{{{\rm{i}}}}}}}}}^{+})g({t}_{{{{{{{{\rm{c}}}}}}}}}^{+}){e}^{-({t}_{{{{{{{{\rm{c}}}}}}}}}^{+}-{t}_{{{{{{{{\rm{i}}}}}}}}}^{+})/{T}_{2}}{e}^{i\omega {t}_{{{{{{{{\rm{c}}}}}}}}}^{+}-iS({t}_{{{{{{{{\rm{i}}}}}}}}}^{+},{t}_{{{{{{{{\rm{c}}}}}}}}}^{+})}\mathop{\sum }\limits_{m=0}^{N-1}{e}^{im\omega {T}_{0}}$$

8 $${j}_{{{{{{{{\rm{inter}}}}}}}}}^{-}(\omega )=\omega F({t}_{{{{{{{{\rm{i}}}}}}}}}^{-})g({t}_{{{{{{{{\rm{c}}}}}}}}}^{-}){e}^{-({t}_{{{{{{{{\rm{c}}}}}}}}}^{-}-{t}_{{{{{{{{\rm{i}}}}}}}}}^{-})/{T}_{2}}{e}^{i\omega {t}_{{{{{{{{\rm{c}}}}}}}}}^{-}-iS({t}_{{{{{{{{\rm{i}}}}}}}}}^{-},{t}_{{{{{{{{\rm{c}}}}}}}}}^{-})}\mathop{\sum }\limits_{m=0}^{N-1}{e}^{im\omega {T}_{0}}$$

and their interference yields the harmonic intensity,

9 $${I}_{\omega }=|{j}_{{{{{{{{\rm{inter}}}}}}}}}^{+}(\omega )+{j}_{{{{{{{{\rm{inter}}}}}}}}}^{-}(\omega ){|}^{2}\propto {\left[\frac{\sin (N\omega {T}_{0}/2)}{N\sin (\omega {T}_{0}/2)}\right]}^{2}{\sin }^{2}\left(\frac{\omega {{\Delta }}{t}_{{{{{{{{\rm{c}}}}}}}}}-{{\Delta }}S}{2}\right)$$

here *ω* is the harmonic frequency, *T*_0_ and *ω*_0_ are the MIR period and frequency. ${{\Delta }}{t}_{{{{{{{{\rm{c}}}}}}}}}={t}_{{{{{{{{\rm{c}}}}}}}}}^{+}-{t}_{{{{{{{{\rm{c}}}}}}}}}^{-}$ and ${{\Delta }}S=S({t}_{{{{{{{{\rm{i}}}}}}}}}^{+},{t}_{{{{{{{{\rm{c}}}}}}}}}^{+})-S({t}_{{{{{{{{\rm{i}}}}}}}}}^{-},{t}_{{{{{{{{\rm{c}}}}}}}}}^{-})$. In the absence of the THz field, Δ*t*_c_ = 0.5*T*_0_ and Δ*S* = 0. The first term of Eq. 9, the *N*-cycle interference factor, restricts the frequencies of the emitted light to be integer multiplies (the harmonics) of the fundamental frequency. The second term, the up-down interference factor, contains information of how the THz field affects the dipole phase. We define *δϕ* as the extra dipole phase difference (between the up and down trajectories) between the with and without THz field cases,

10 $$\delta \phi={{\Delta }}\phi -{{\Delta }}{\phi }_{0}=(\omega {{\Delta }}{t}_{{{{{{{{\rm{c}}}}}}}}}-{{\Delta }}S)-\omega*0.5{T}_{0}$$

and the even-/odd-order harmonic intensities are proportional to,

11 $${I}_{2n}\propto {\sin }^{2}\frac{\delta {\phi }_{2n}}{2}={\sin }^{2}\frac{2n{\omega }_{0}({{\Delta }}{t}_{{{{{{{{\rm{c}}}}}}}}}-0.5{T}_{0})-{{\Delta }}S}{2}$$

12 $${I}_{2n+1}\propto {\cos }^{2}\frac{\delta {\phi }_{2n+1}}{2}={\cos }^{2}\frac{(2n+1){\omega }_{0}({{\Delta }}{t}_{{{{{{{{\rm{c}}}}}}}}}-0.5{T}_{0})-{{\Delta }}S}{2}$$

For a first order approximation, both Δ*t*_c_ and Δ*S* are proportional to the THz field strength. Therefore, within the small angle approximation of Eqs. 11 and 12, the even-/odd-order harmonic yields increase/decrease linearly with the THz intensity.

In the semiclassical analysis, we can calculate the short and long electron trajectories separately. Figure 6 shows a typical calculation. The small angle approximation holds for short trajectories. Whereas for long trajectories, the absolute values of both *ω*(Δ*t*_c_ − 0.5*T*_0_) and −Δ*S* are larger than those for short trajectories, and *δϕ* could easily exceed *π* with THz field strengths on the order of a few hundred kV cm^−1^, which breaks the small angle approximation and results in oscillations of the harmonic yield as a function of the THz intensity. The order-dependence of the odd-order harmonic yield modulations are also very different for harmonics generated from the short and long trajectories (Fig. 5).

In the end, we discuss the influence of the THz field to the excitation process. Depends on the polarity of the THz field relative to that of the MIR field, the application of the THz field either enhances (same polarity) or suppresses (opposite polarity) the photocarrier excitation, therefore introduces an imbalance in the amplitude of the interband currents between adjacent MIR half-cycles. In ref. ^33^, such imbalance of ionization/excitation is considered as a modulation of the imaginary part of the dipole phase whereas the imbalance during propagation (that we discussed throughout the manuscript) is a modulation of the real part of the dipole phase. Following the same analysis, we can include the THz induced imbalance of excitation by adding a pre-factor to the interband currents, i.e., ${J}_{inter}^{\pm }={e}^{\pm {{{{{{{\rm{Im}}}}}}}}(\delta \phi )}\cdots$ in Eqs. 7 and 8. Accordingly, Eqs. 11 and 12 become,

13 $${I}_{2n}\propto \frac{1}{2}\left(\cosh [2{{{{{{{\rm{Im}}}}}}}}(\delta {\phi }_{2n})]-\cos [{{{{{\mathrm{Re}}}}}} (\delta {\phi }_{2n})]\right)\simeq {\sin }^{2}\frac{{{{{{\mathrm{Re}}}}}} (\delta {\phi }_{2n})}{2}+{[{{{{{{{\rm{Im}}}}}}}}(\delta {\phi }_{2n})]}^{2}$$

14 $${I}_{2n+1}\propto \frac{1}{2}\left(\cosh [2{{{{{{{\rm{Im}}}}}}}}(\delta {\phi }_{2n+1})]+\cos [{{{{{\mathrm{Re}}}}}} (\delta {\phi }_{2n+1})]\right)\simeq {\cos }^{2}\frac{{{{{{\mathrm{Re}}}}}} (\delta {\phi }_{2n+1})}{2}+{[{{{{{{{\rm{Im}}}}}}}}(\delta {\phi }_{2n+1})]}^{2}$$

Since the THz field is a lot weaker than the MIR field, the THz induced imbalance in the amplitude of the interband currents is small. In fact, we can extract from SBEs simulation that ${{{{{{{\rm{Im}}}}}}}}(\delta \phi ) \sim 0.04$, hence ${[{{{{{{{\rm{Im}}}}}}}}(\delta \phi )]}^{2} \sim 0.16\%$. Therefore, for the above-band gap harmonic yield modulation (>10% for odd-orders), we can ignore the influence of the THz field to the excitation process.

## Supplementary information

Supplementary Information

Peer review file

## Data Availability

All data that support the plots within this manuscript are available upon request. Source data for Figs. 2–6 in the main manuscript are provided with this paper. Source data are provided with this paper.

## Source data

Source Data

## Code availability

The codes that run the semiconductor Bloch simulations and semiclassical calculations are available upon reasonable request.
