# Supplementary material for: High-order harmonic generation from a thin film crystal perturbed by a quasi-static terahertz field
Source: Nat Commun. 2023 May 5;14:2603. doi: 10.1038/s41467-023-38187-0 (PMC10163039; doi:10.1038/s41467-023-38187-0)
Supplement: Supplementary file 1 — Supplementary Information [file 41467_2023_38187_MOESM1_ESM.pdf]

High-Order Harmonic Generation from a Thin Film Crystal Perturbed by a Quasi-Static Terahertz Field
Supplementary Information

Sha Li, Yaguo Tang, Lisa Ortmann, Bradford K. Talbert, Cosmin I. Blaga, Yu Hang Lai, Zhou Wang, Yang Cheng, Fengyuan Yang, Alexandra S. Landsman, Pierre Agostini, and Louis F. DiMauro

Department of Physics, The Ohio State University, Columbus, Ohio 43210, USA

CONTENTS

1. Comparison between the the parallel and perpendicular MIR-THz polarization configurations 2
2. Probing the band structure via THz-dressed HHG 6
3. THz metrology: EOS versus HHG 7
4. Simulation based on semiconductor Bloch model: Results 10
5. Even-order harmonic frequency shift: Extended study 18

References 23

1. Comparison between the the parallel and perpendicular MIR-THz polarization configurations

In ref. [1] it has been shown that for bulk silicon (Si) crystal, there is a factor of 3 difference in the even-order harmonic conversion efficiency between the parallel and perpendicular MIR-THz polarization configurations, which agrees with perturbative calculation based on values of the $\chi^{(3)}$ tensor. In our study, calculation based on $\chi^{(3)}$ of ZnO suggests that there should be a factor of 10 difference, which deviates from the experimental observation (figures 1). This further confirms the non-perturbative HHG process under our experimental conditions.

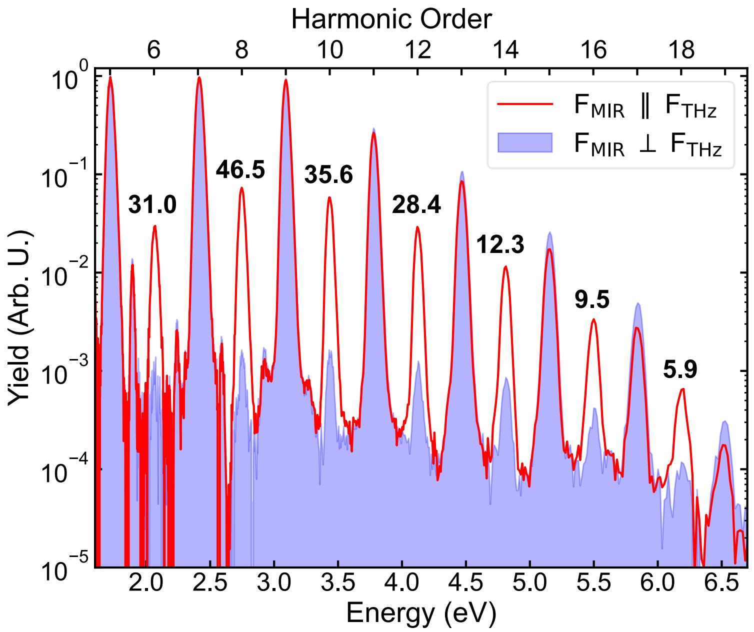

FIG. 1: HHG spectrum under the two configurations of $F_{\mathrm{MIR}} \parallel F_{\mathrm{THz}}$ and $F_{\mathrm{MIR}} \perp F_{\mathrm{THz}}$ at the same MIR and THz intensities. The inset numbers are the ratios of each even-order harmonic yield between these two configurations.

We also find an interesting feature for the modulation of the total harmonic yield (Y_{tot}): When the MIR and THz fields are parallel, the generation/suppression of even-/odd-order harmonics are similar in amplitude (main text figure 2b), and Y_{tot} always increases on the order of 1-2% (figure 3b, numbers). Whereas with orthogonally polarized MIR and THz fields, the suppression of odd-order harmonics is slightly stronger than the generation of even-orders (figure 2a, inset), and Y_{tot} decreases on the order of 1% (figure 2b, numbers).

These are consistent with a generalized recollision HHG process: With the THz perturbing field parallel to the MIR drive, the total peak field slightly increases, leading to a very small increase in the excitation and therefore an enhancement of Y_{tot}. Whereas with orthogonally polarized MIR and THz fields, the transverse displacement of the electron driven by the THz field reduces the electron-hole recollision probability, therefore Y_{tot} is suppressed.

In ref. [2], the relative even-order harmonic yields from an orthogonal ω-2ω drive have been used to extract the HHG recollision angles. It was shown that (for short trajectories) electrons associate with lower-order harmonics experience larger recollision angles, as they are "born" when the fundamental field is weak and are affected more by the second harmonic perturbing field. Following the same argument, we calculate the recollision angles with orthogonal MIR-THz two-color field from single-shot measurement of the harmonic spectrum (figure 2b). Our results distinct from ref. [2] that the recollision angle increases with the harmonic order, implying that electrons associate with higher-order harmonics spend more time in "continuum" and drift more in the transverse direction due to the THz drive. This clearly indicate that the second harmonic and the THz perturbing fields fall in different Keldysh scaling regimes.

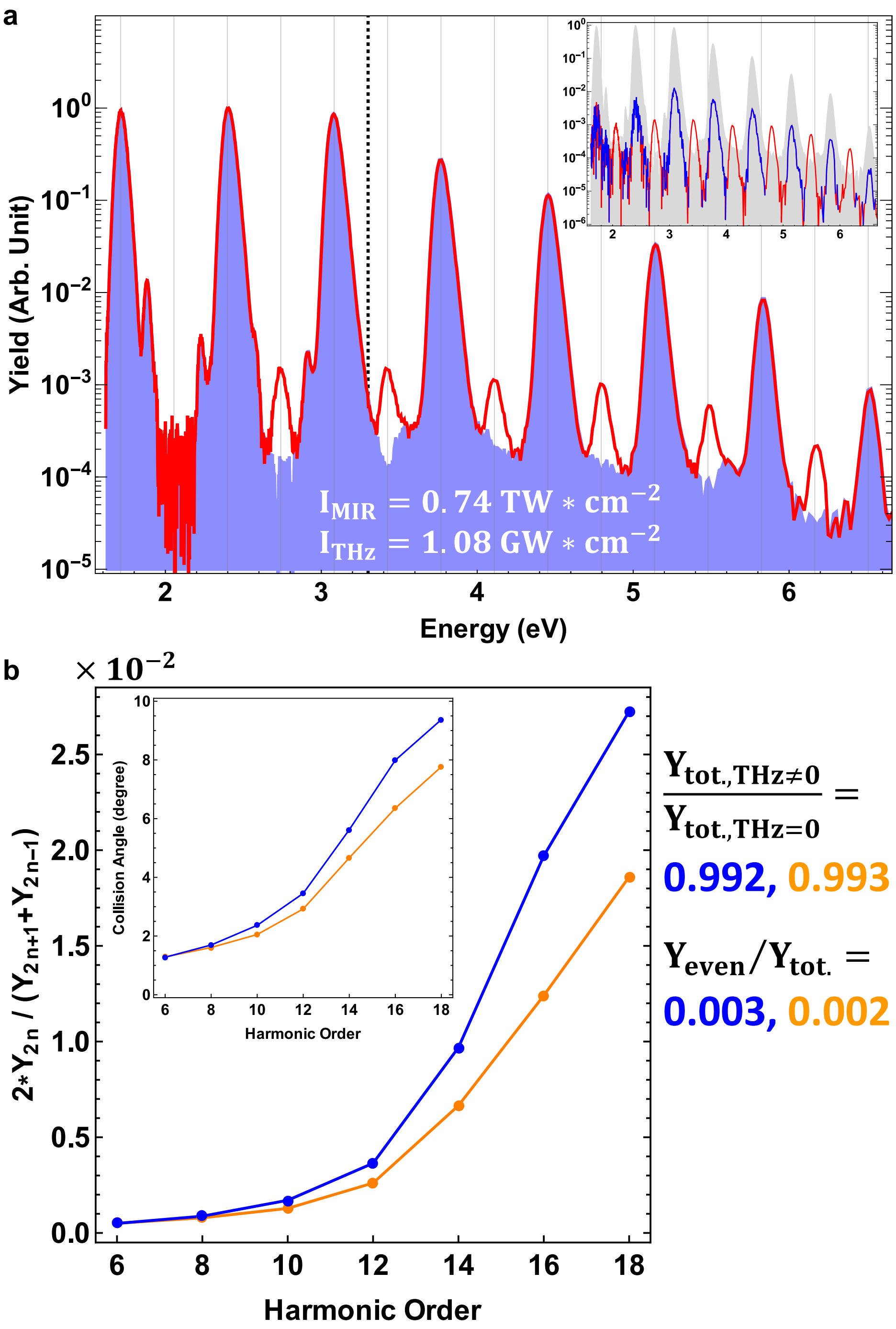

FIG. 2: Modulation of the high harmonic distribution with orthogonal MIR-THz two-color field. **(a)** HHG spectrum without (shaded blue) and with (solid red) the THz field. **Inset:** The difference spectrum (same as main text figure 2b). **(b)** Even-order harmonic yield relative to its adjacent odd-orders, $2 * Y_{2n}/(Y_{2n-1} + Y_{2n+1})$, as a function of the harmonic order at a fixed THz intensity $I_{\mathrm{THz}} = 1.08$ GW $* \mathrm{cm}^{-2}$ and $I_{\mathrm{MIR}} = 0.41$ (blue), 0.74 (orange) TW $* \mathrm{cm}^{-2}$, respectively. **Inset**: Electron-hole recollision angle calculated by $\theta_{2n} = \arctan\sqrt{2 * Y_{2n}/(Y_{2n+1} + Y_{2n-1})}$.

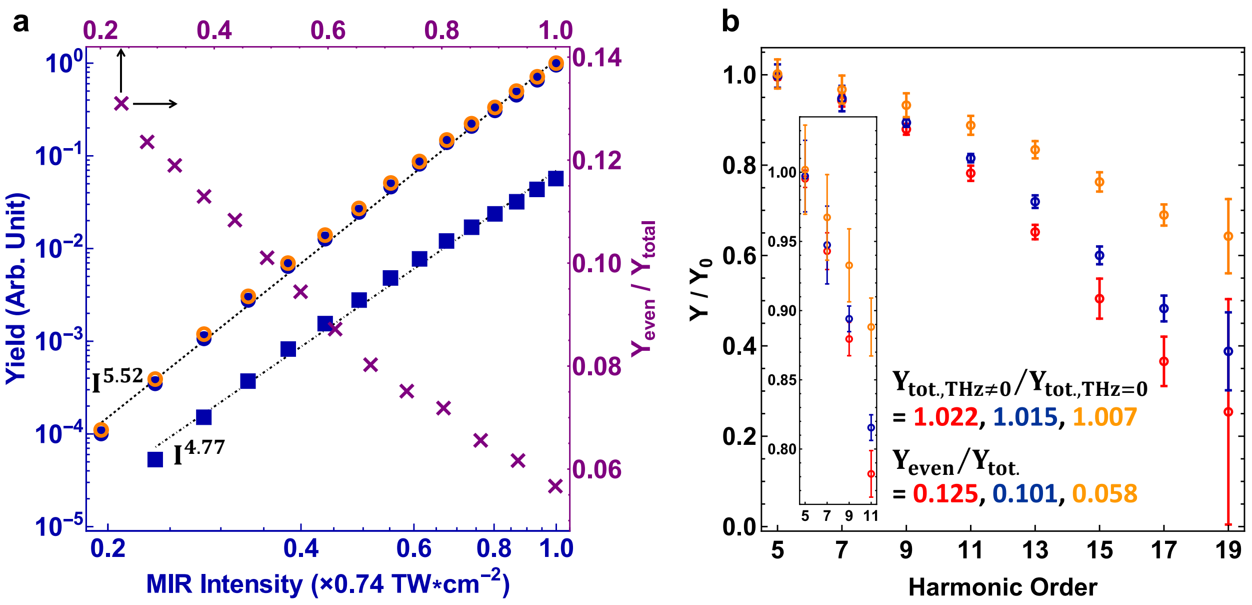

FIG. 3: With parallel MIR-THz two-color field, **(a)** Harmonic yield as a function of the MIR intensity at a fixed $I_{\mathrm{THz}} = 1.08$ GW$*$cm^{-2}: total (from the 5th to the 19th orders) odd-orders without the THz field (open orange circle) and with the THz field (filled blue circle, and dashed line is linear fit to data), total even-orders with the THz field (filled blue square, and dash-dotted line is linear fit to data), total even-orders to total harmonic yield ratio, *i.e.* the total even-order harmonic conversion efficiency, as a function of the MIR intensity on linear-linear scale (purple cross). **(b)** The relative odd-order harmonic yield modulation, Y/Y_0, here Y and Y_0 are respectively the yields with and without the THz field, as a function of the harmonic order, at $I_{\mathrm{THz}} = 1.08$ GW $*$ cm^{-2} and $I_{\mathrm{MIR}} = 0.28$ (red), 0.41 (blue), 0.74 (orange) TW $*$ cm^{-2}, respectively. Error bars are standard deviations of 10 measurements.

2. Probing the band structure via THz-dressed HHG

Our semiclassical analysis of the electron motion during the generalized recollision HHG process shows that the THz field induces a first-order perturbation to the electron dipole phase difference $\delta\phi = \omega(\Delta t_c - 0.5T_0) - \Delta S \propto F_{\mathrm{THz}}$, and the odd-order harmonic yield follows a $\cos^2(\delta\phi/2)$ suppression. Since the equation of motion of the electron is directed related to the reciprocal space group velocity (the band dispersion of the crystal), the band structure information is imprinted on the relationship between $\delta\phi$ and the harmonic order.

To verify the ability to probe the band structure, we perform semiclassical calculations for the top three valence bands: heavy holes, light holes and split-off [3]. These three bands have very close energies at Γ point, but different band dispersions lead to different electron trajectories after the excitation. Figure 4 shows calculations of the THz induced harmonic yield modulation: For short trajectories, the split-off band deviates more and more from the heavy and light holes bands as the harmonic order increases. For long trajectories, the order-dependence of the modulation is very sensitive to the band structure.

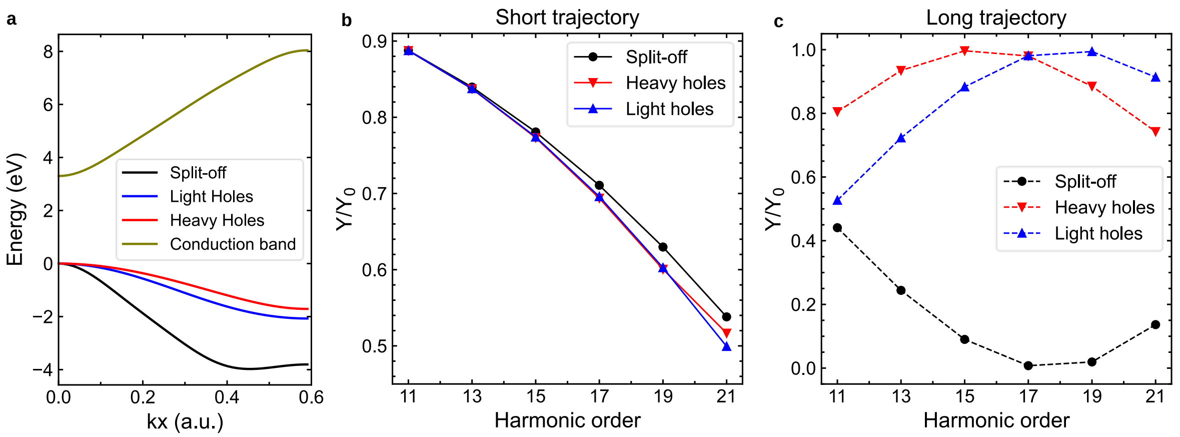

FIG. 4: Comparisons of the heavy holes, light holes and split-off valance bands of the THz induced odd-order harmonic yield modulation. **(a)** The band dispersion along the Γ-M direction from the nonlocal semi-empirical pseudopotential calculation, from ref. [3]. Semiclassical calculations for short **(b)** and long **(c)** trajectories. $I_{\mathrm{MIR}} = 0.74$ TW $*$ cm^{-2} and $I_{\mathrm{THz}} = 1.08$ GW $*$ cm^{-2} are used in the calculations.

3. THz metrology: EOS versus HHG

Figure 5 plots the individual harmonic yield as a function of the MIR and THz intensities. As is shown, from the 5th- to the 19th-orders, all harmonic yields scale linearly with the THz intensity, this linear dependence could be utilized for precise THz metrology. In figure 6, we measure the individual harmonic yield as a function of the MIR-THz field delay, which shows that all harmonic orders are in phase, further supports that the harmonic yield modulation responds to the instantaneous THz intensity and sub-cycle dynamics have been washed out on the ps THz period time scale. We also compare the THz field sampled by electro-optic sampling (EOS), SHG, and HHG (figure 6c). Though we do not draw the conclusion from the comparison that the HHG method has better resolution, as our experimental setup is not optimized for EOS, we do provide the following arguments that the THz-dressed HHG has the potential for developing ultra-broadband, ultra-sensitive THz time domain spectroscopy (THz-TDS) [4]: Conventional EOS has bandwidth limit because the EO coefficient of the sampling crystal is frequency dependent and may vary a lot within the THz frequency range. For example, ZnTe is typically good for frequencies below 3 THz. If we use high-order harmonics to sample the THz field, such bandwidth limit is released. In addition, since the effective interaction length for (above-band gap) HHG is ultra-thin, typically tens of nm, walk-off effect due to velocity mismatching between the THz and the probe pulses is eliminated. Besides, the highly nonlinear nature of the HHG process effectively shortens the probe pulse duration, resulting in an increased temporal resolution. Last but not least, EOS typically uses crystals with large χ^2 to achieve strong Pockels effect, whereas in principle, any crystal can realize HHG based THz field sampling. However, since the harmonic yield directly samples the THz intensity, careful design is needed to determine the polarity of the THz field. For example, a DC bias can be applied, or χ^2 crystals that can generate even-order harmonics in the absence of the THz field can be used.

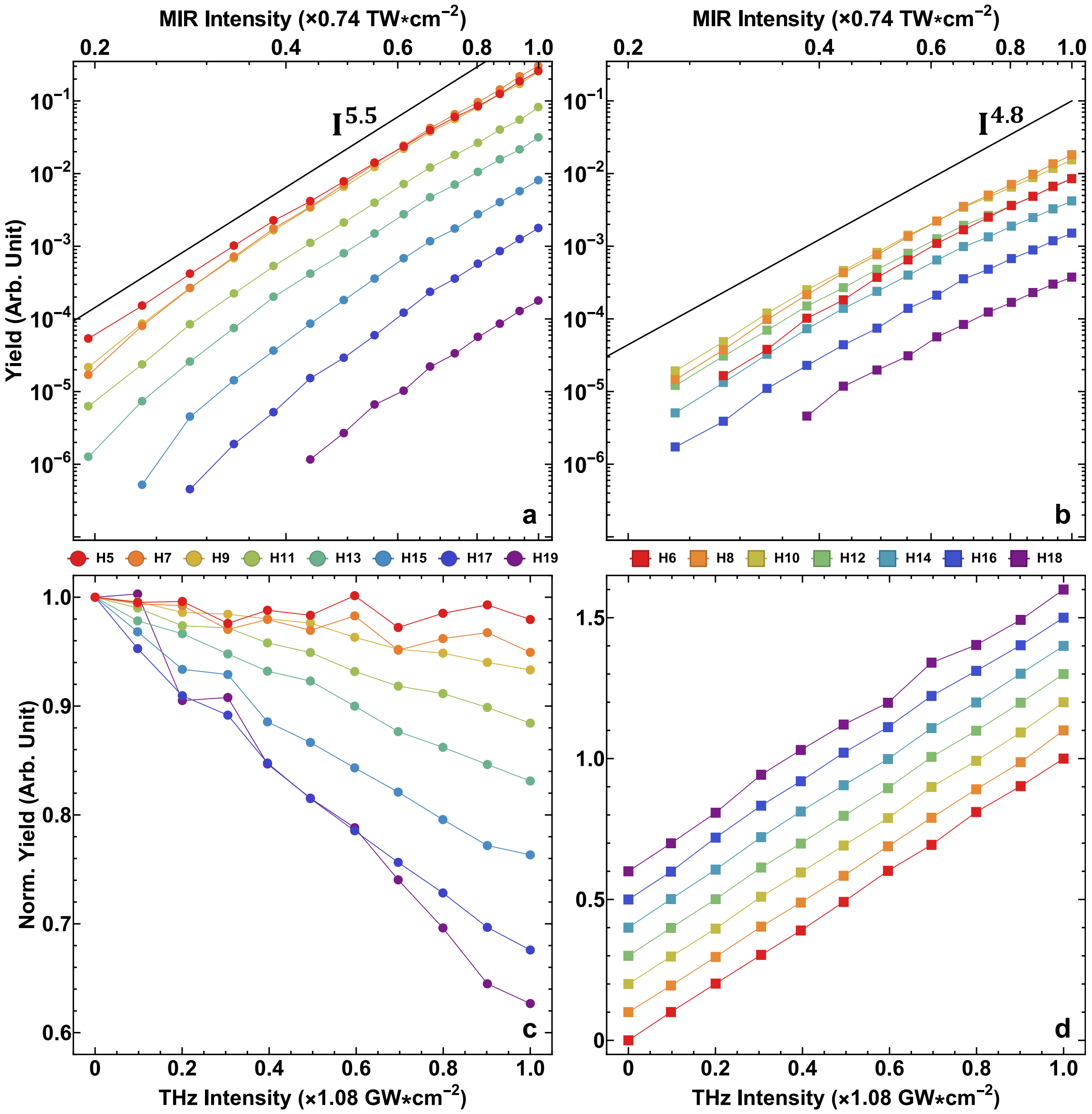

FIG. 5: From the 5th to the 19th order harmonics: **(a-b)** Harmonic yield as a function of the MIR intensity at a fixed THz intensity $I_{\mathrm{THz}} = 1.08\ \mathrm{GW} * \mathrm{cm}^{-2}$. Solid lines are power scale guidelines. **(c-d)** Normalized harmonic yield as a function of the THz intensity at a fixed MIR intensity $I_{\mathrm{MIR}} = 0.74\ \mathrm{TW} * \mathrm{cm}^{-2}$. Each harmonic order is individually normalized to their maximum. In **(d)** the harmonic yields are y-offset for clearer visualization.

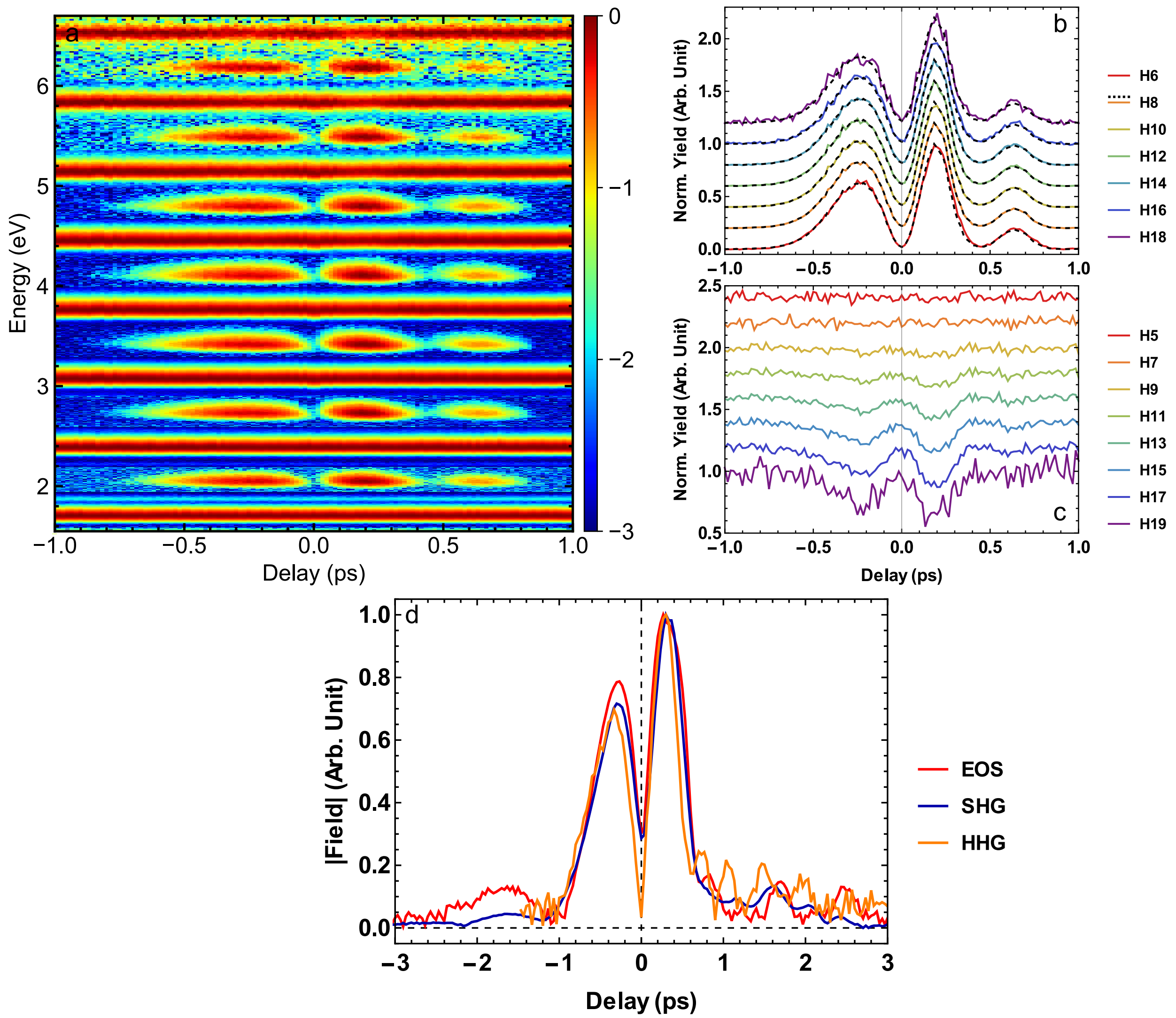

FIG. 6: THz methrology based on HHG. High-order harmonic spectrogram **(a)** and integrated even-order **(b)** and odd-order **(c)** harmonic yield, as a function of the delay between the MIR and THz pulses. Each harmonic order is individually normalized. In **b** and **c**, The y-axis is offset for clearer visualization. **(d)** Back-to-back comparison of THz field (absolute value) sampled by EOS (red): 200 μm ZnTe as the sampling crystal, and 800 nm pulse as the probe. SHG (blue): 200 μm ZnTe as the sampling crystal, and 800 nm pulse as the fundamental drive. HHG (orange): 100 nm ZnO as the sampling crystal, and 3.6 μm pulse as the fundamental drive, and the 14th-order harmonic is used. Note the slight difference in the THz waveform between **a-c** and **d** is due to the experiments been performed at different days.

4. Simulation based on semiconductor Bloch model: Results

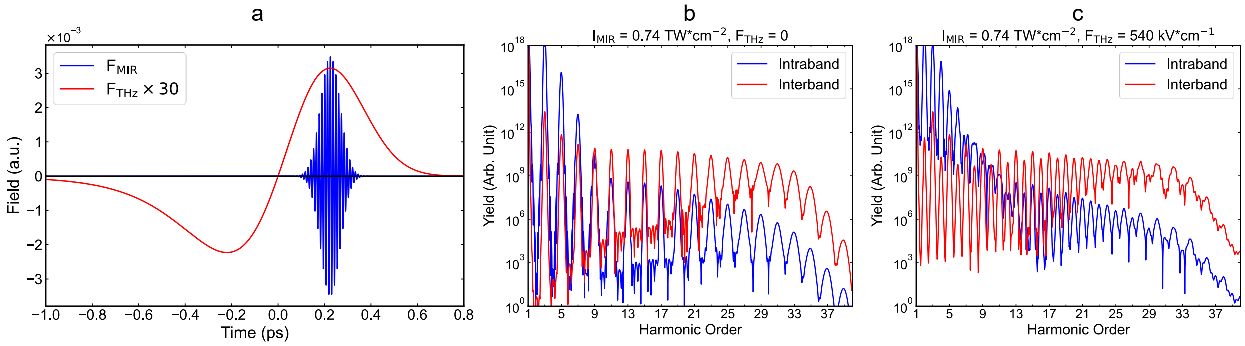

FIG. 7: **(a)** Temporal profiles of the MIR and THz pulses used in the SBEs simulation. Typical HHG spectra without **(b)** and with **(c)** the THz field from the SBEs simulation.

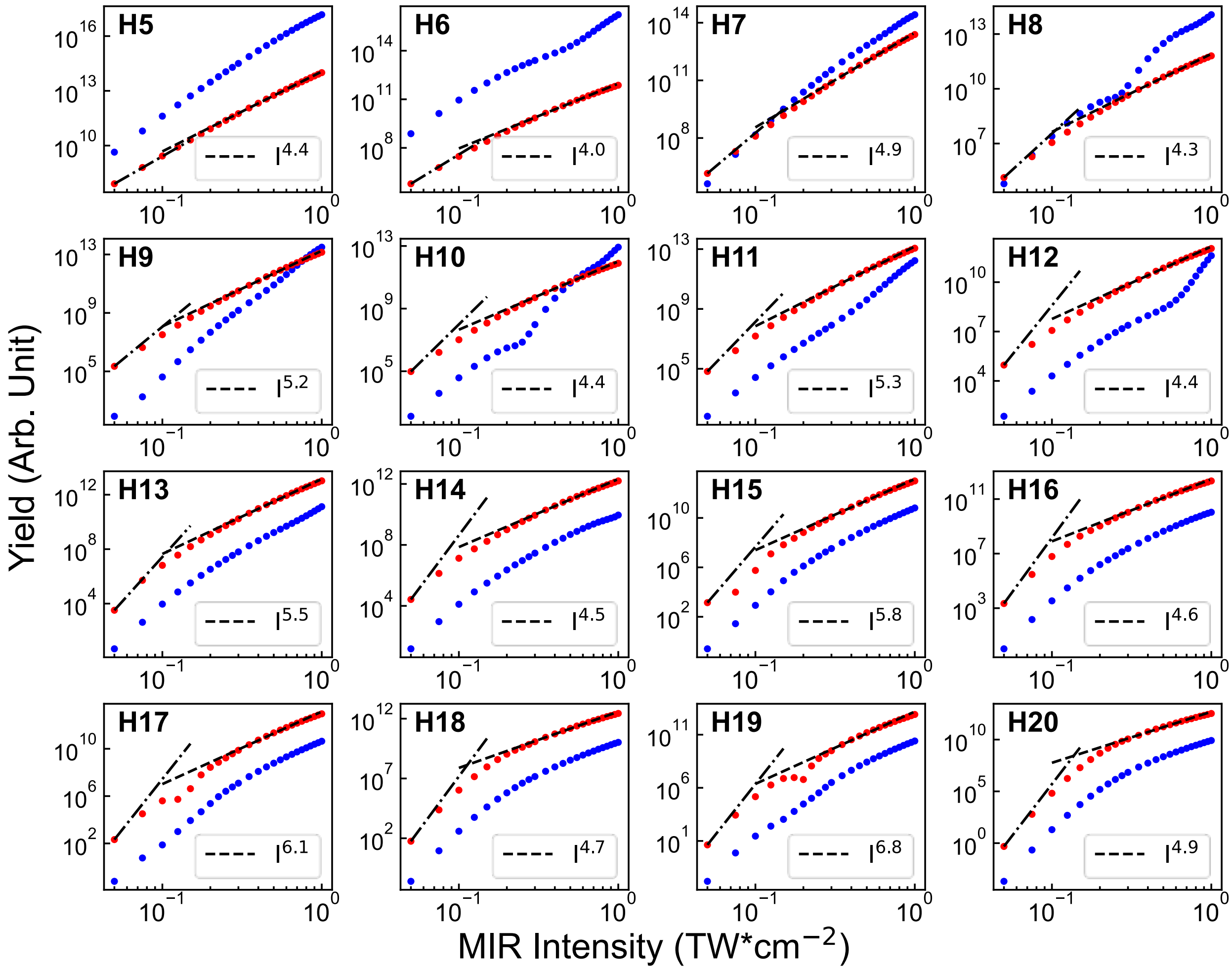

FIG. 8: Interband (red) and Intraband (blue) harmonic yield as a function of the MIR intensity, at $I_{\mathrm{THz}} = 1.08$ GW $*$ cm^{-2}. Dashed line is linear fit to interband yield in the MIR intensity regime $I_{\mathrm{MIR}} = 0.3$-0.8 TW $*$ cm^{-2}. Dash-dotted line is multiphoton perturbative scaling ($I_{q\omega} \propto I_{\omega}^{q}$) guideline.

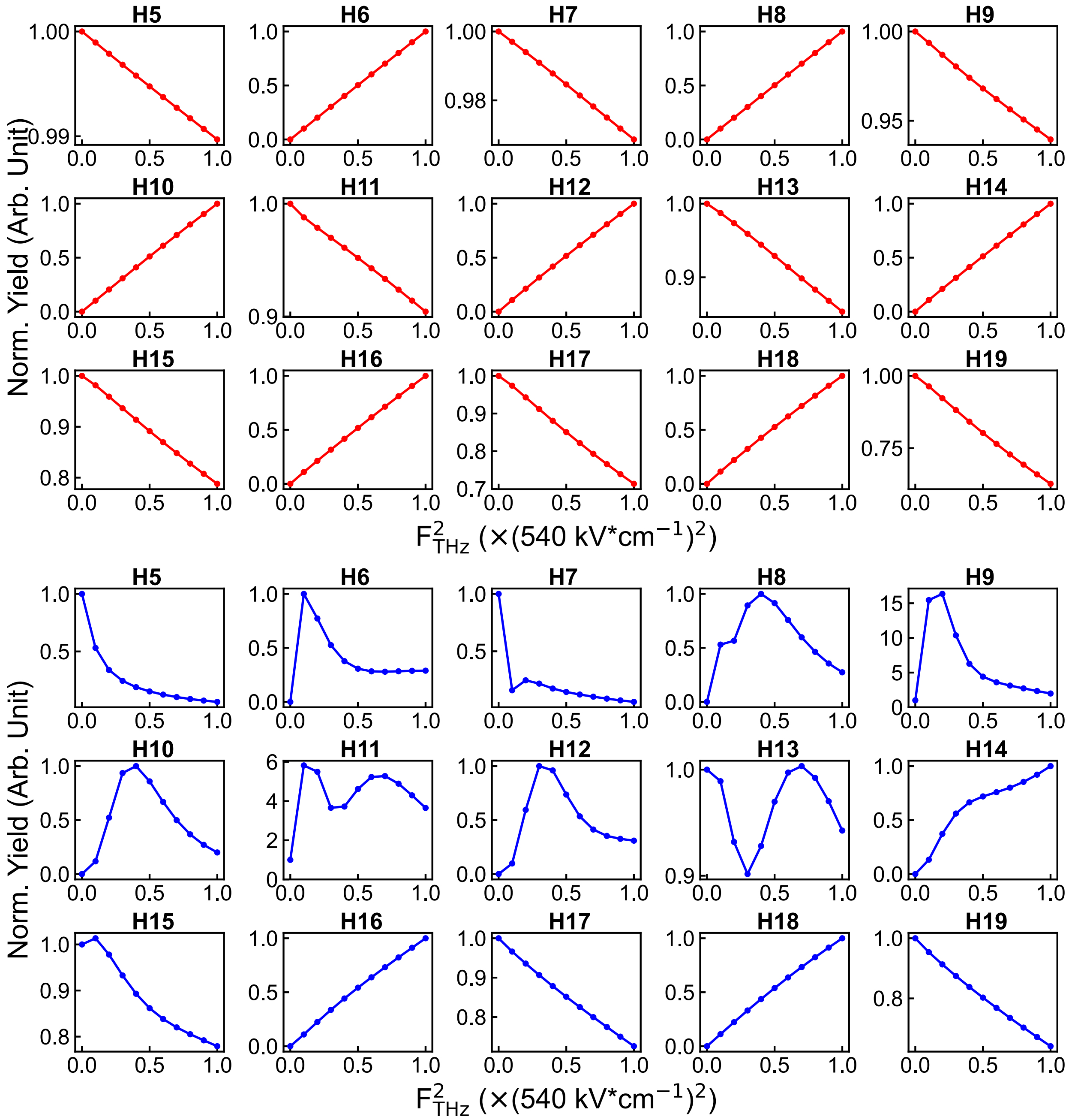

FIG. 9: Normalized interband (red) and intraband (blue) harmonic yield as a function of the THz intensity, at $I_{\mathrm{MIR}} = 0.74\ \mathrm{TW} * \mathrm{cm}^{-2}$. Even-order harmonics are normalized to their maximum values. Odd-order harmonics are normalized to their values without the THz field.

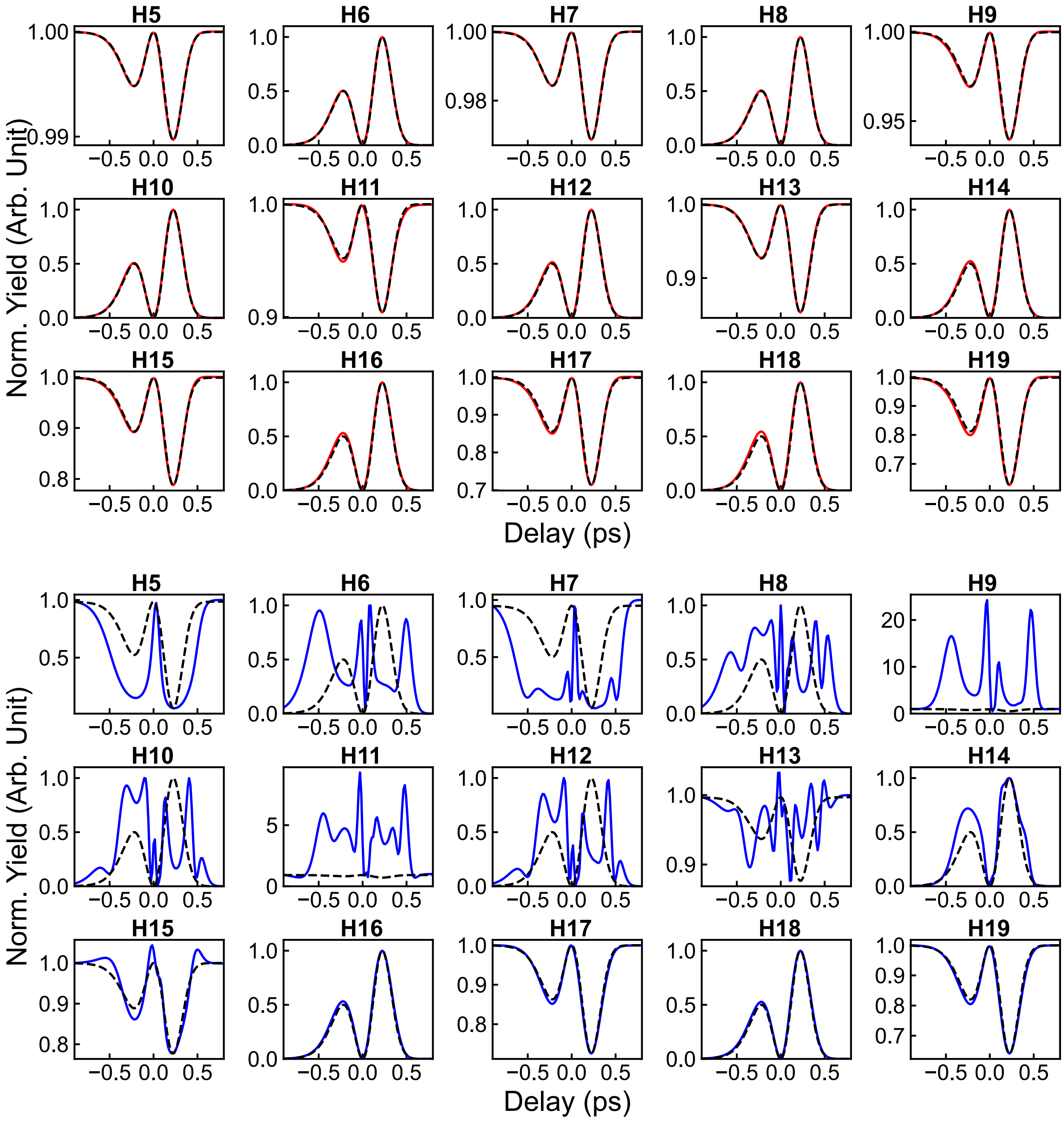

FIG. 10: Normalized interband (red) and intraband (blue) harmonic yield as a function of the MIR-THz field delay, at $I_{\mathrm{MIR}} = 0.74\ \mathrm{TW} * \mathrm{cm}^{-2}$ and $I_{\mathrm{THz}} = 1.08\ \mathrm{GW} * \mathrm{cm}^{-2}$. Black dashed line shows the temporal profile of the THz intensity. Even-order harmonics are normalized to their maximum values. Odd-order harmonics are normalized to their values without the THz field.

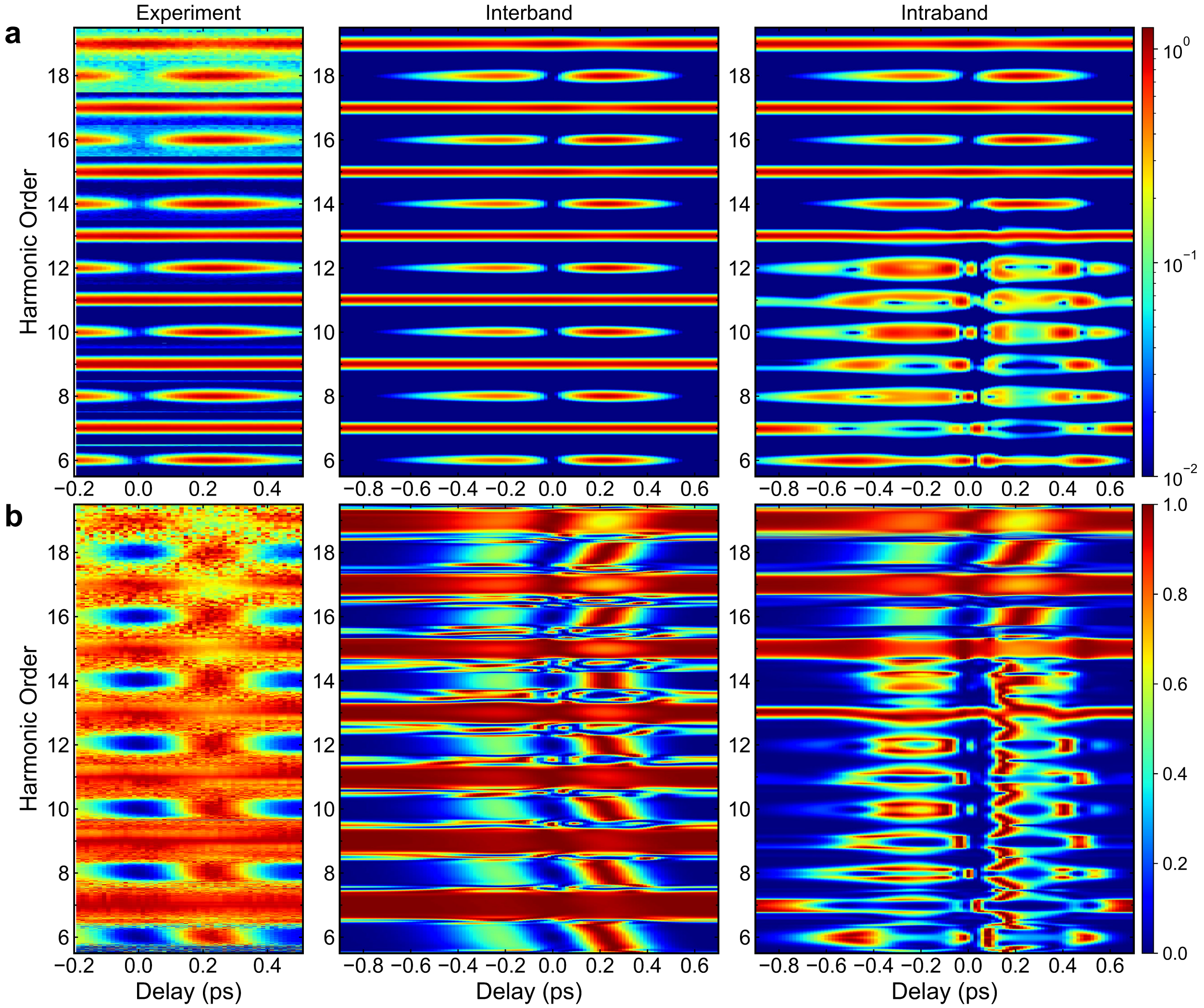

FIG. 11: Normalized harmonic spectrogram as a function of the MIR-THz field delay, at $I_{\mathrm{MIR}} = 0.74\ \mathrm{TW} * \mathrm{cm}^{-2}$ and $I_{\mathrm{THz}} = 1.08\ \mathrm{GW} * \mathrm{cm}^{-2}$. Each harmonic order is normalized to their maximum **(a)**. Each energy pixel "row" is normalized to their maximum **(b)**.

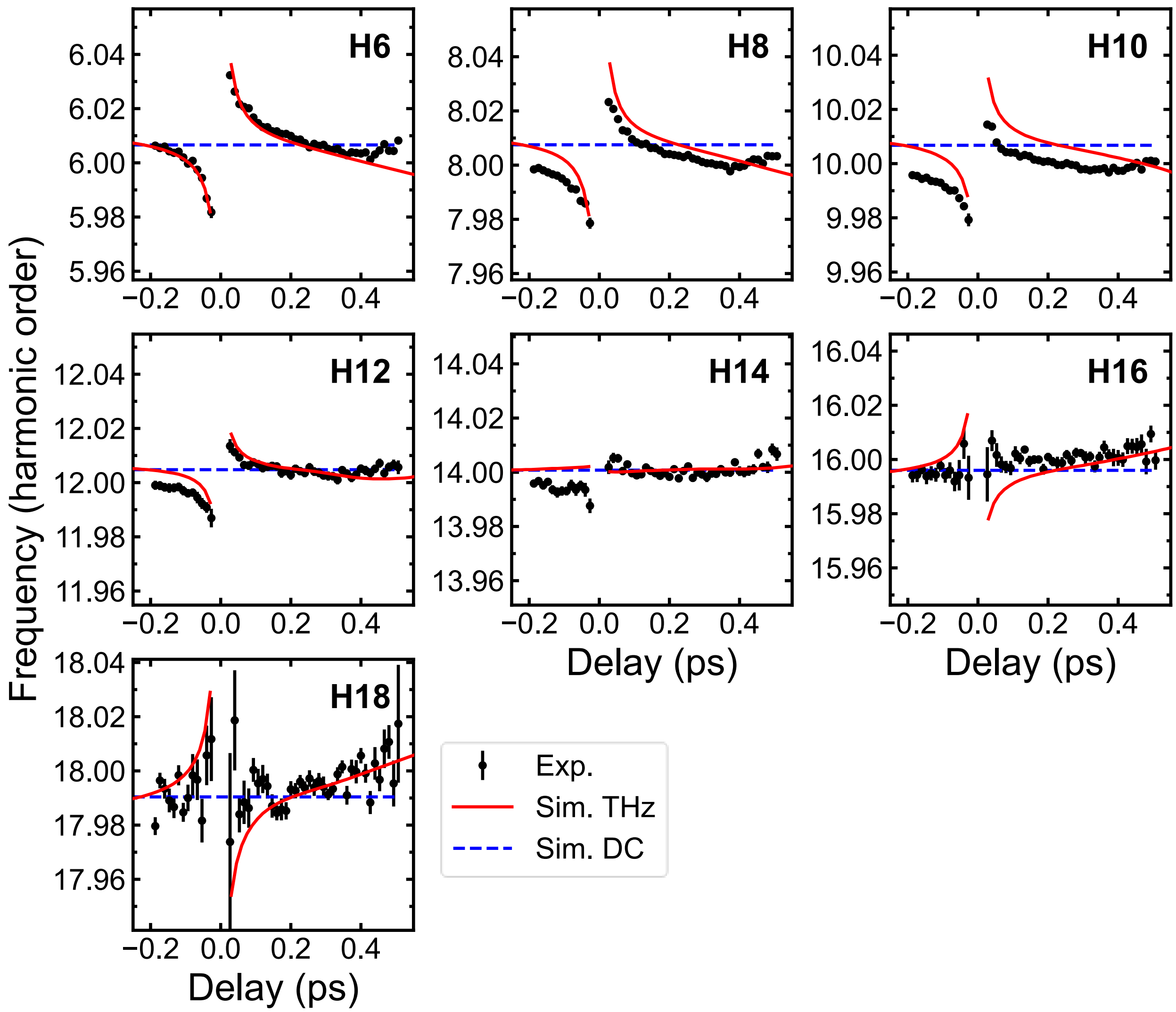

FIG. 12: From the 6th to the 18th even-order harmonics, centro-frequency of the harmonic peak as a function of the MIR-THz field delay.

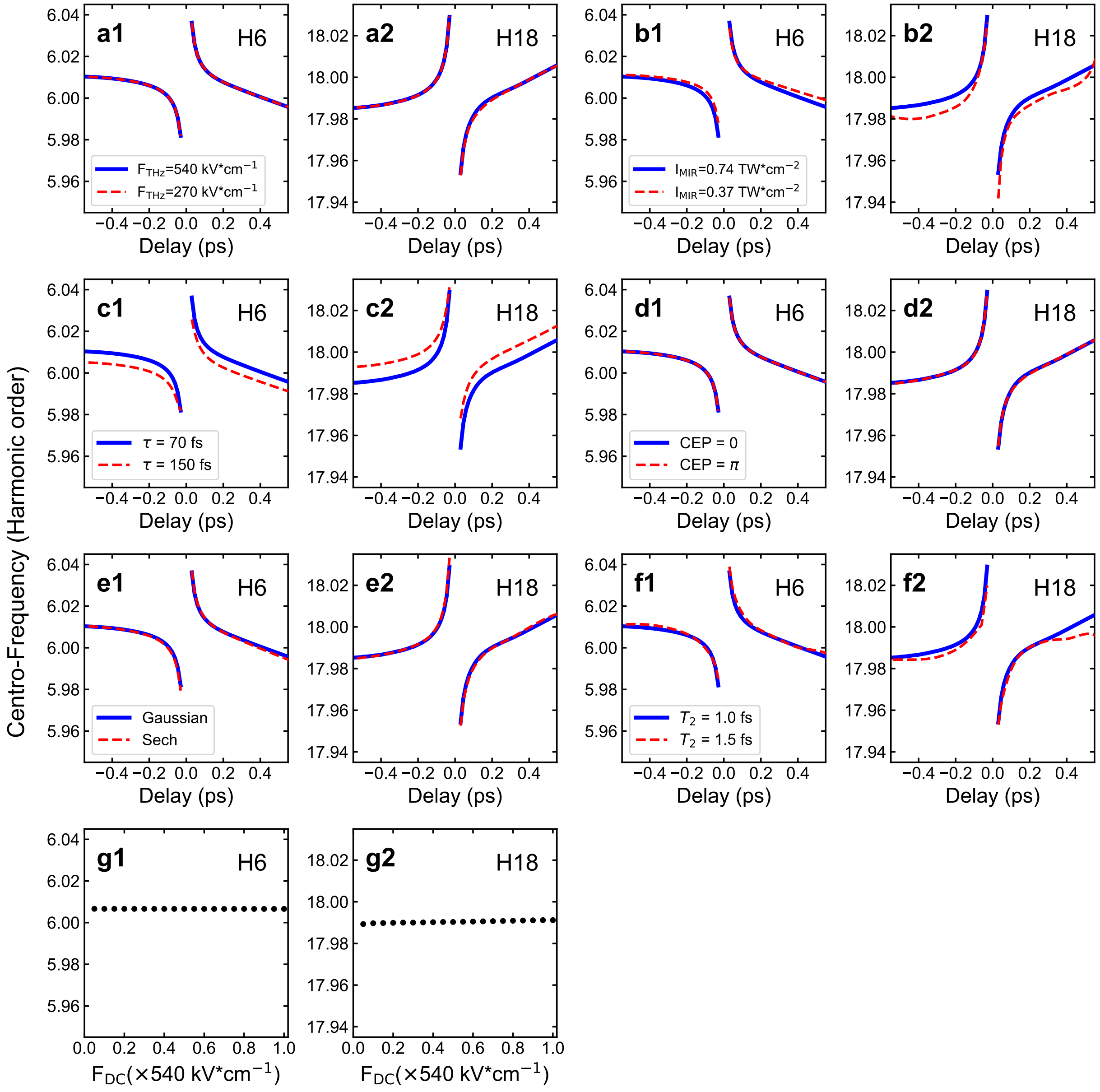

FIG. 13: For the 6th and the 18th order harmonics, the frequency shift under different SBEs simulating conditions by varying: the THz intensity **(a)**, the MIR intensity **(b)**, the MIR pulse duration **(c)**, the MIR pulse CEP **(d)**, the MIR pulse shape **(e)**, the dephasing time **(f)**, the field strength of a static perturbing field **(g)**.

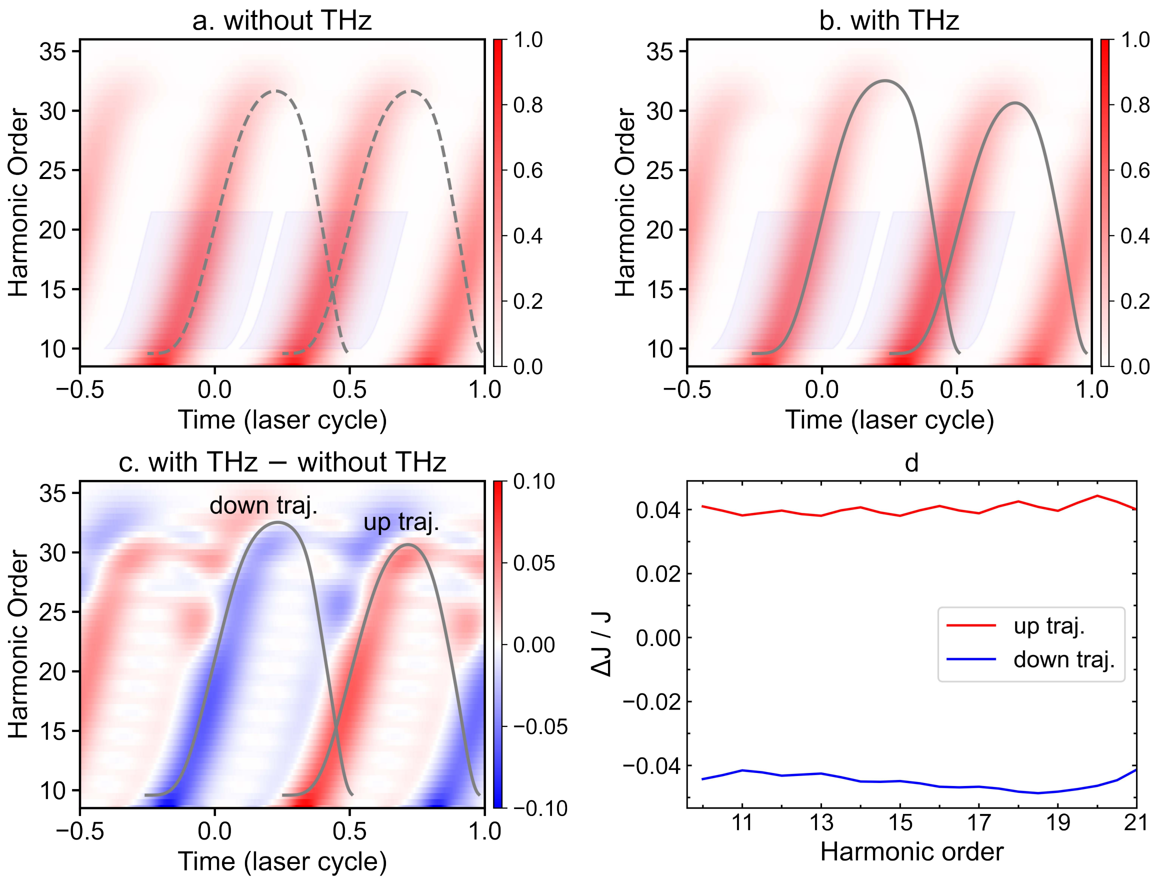

FIG. 14: Time-frequency spectrogram (linear color-scale) of the harmonic yield near the peak of the MIR pulse from the SBEs interband simulation: without the THz field **(a)**, with the THz field **(b)**, and their difference **(c)**. Harmonics associate with long electron trajectories have been eliminated due to ultrafast dephasing. The dashed/solid grey curves are semiclassical calculation of the harmonic emission times without/with the THz field, respectively. **(d)** Relative amplitude modulation of the interband current, $\Delta J/J = (J_{\text{with THz}} - J_{\text{without THz}})/J_{\text{without THz}}$, as a function of the harmonic order. The yield (Y) for each harmonic order is summed over a half-cycle temporal window (indicated by the background blue windows in **a** and **b**), and $J = \sqrt{Y}$.

5. Even-order harmonic frequency shift: Extended study

To unravel more the delay-dependent even-order harmonic frequency shift, we perform SBEs simulations to investigate its sensitivity to the crystal band gap and the MIR characteristics: wavelength, short pulse carrier-envelope-phase (CEP) and group delay dispersion (GDD). The results are summarized in figures 15-19. Unless otherwise specified, default simulation parameters are $\epsilon_g = 3.3$ eV, $\lambda = 3.6$ μm, $\tau_{FWHM} = 70$ fs, CEP = 0 and GDD = 0.

In the fitting equation, $\omega_{2n}/\omega_0 = C_0 + C_1 * F'_{\mathrm{THz}}(t)/F_{\mathrm{THz}}(t)$, the frequency offset ($C_0 - 2n$) and delay-dependent shift (C_1) depend on the harmonic order. Interestingly, both parameters approach 0 and reverse sign at around the 14th-order harmonic. SBEs simulations by varying the crystal band gap (while keeping the shape of the band curvature unaltered) suggest that the amplitude and sign of $C_0 - 2n$ and C_1 are sensitive to the width of the band gap (figure 15). This indicates that although the origin of the frequency offset and delay-dependence comes from the MIR field being pulsed and the THz field being non-DC, the crystal band gap also plays an important role in determining the exact value of the emitted harmonic frequencies. To the best of our knowledge, there has been no systematic study about the relationship between the crystal band gap/structure and the harmonic frequency on a fine level and open questions remain. In the future, experimental investigation can be performed with high resolution spectrometer and careful preparation of different crystal samples (*e.g.* thin films to rule out nonlinear distortion and propagation effects). Theoretical verification may be further supported by more “complete” simulation beyond one-dimensional two-band model.

To investigate whether the ratio of the MIR to THz period/wavelength plays a role in the frequency shift, we compare SBEs simulations at two MIR wavelengths, $\lambda = 2.4$ and 3.6 μm, respectively. The results show a MIR wavelength-dependence of C_1 as a function of the harmonic order. However, if we compare at a fixed harmonic photon energy, C_1 only experiences small MIR wavelength-dependence (figure 16), perhaps suggesting that as long as the THz period is much longer than the MIR pulse duration, the frequency shift is not sensitive to the MIR period.

For strong-field light-matter interaction, it is known that as the driving laser pulse duration becomes very short, there will be CEP effects. In our SBEs simulations, we find that

the CEP of the MIR pulse starts to affect the harmonic frequency and the fitting equation $\omega_{2n}/\omega_0 = C_0 + C_1 * F'_{\text{THz}}(t)/F_{\text{THz}}(t)$ breaks down when the (intensity FWHM) MIR pulse duration approaches ∼30 fs, or ∼2.5 cycles (figures 17, 18).

A chirp in the MIR pulse can also introduce harmonic frequency modulations. In the experiment, careful investigations via FROG characterizations, SBEs simulations and MIR-THz delay-scan harmonic spectrum measurements at various MIR pulse GDDs have been performed to ensure that the frequency shift studied in this manuscript does not come from the MIR chirp. Figure 19 compares the experimental measurement with the SBEs simulation the relationship between the MIR GDD and the even-order harmonic frequency shift.

Last but not least, we think more insights between the frequency shift and the HHG process may be unraveled via semiclassical analysis. However, such analysis is extremely challenging and beyond the scope of this work. In the following, we provide some attempt to show the relationship between the slope of the perturbing field and the frequency of the harmonic light. We assume a continuous wave MIR field and a linearly ramped perturbing field $F_l(t) = F_0 + F' * t$, here F_0 is the DC offset and $F' = \mathrm{d}F_l/\mathrm{d}t$ is the ramp rate. Following similar analysis as is used in the METHOD section, within the first order approximation, we can define the recombination times (t_c) and the semiclassical actions (S) for the up ("+") and down ("−") trajectories of the mth MIR field cycle,

$$t^{\pm}_{c,m} = (t^{\pm}_{c,0} + mT_0) \pm \alpha F_l(t = mT_0) = t^{\pm}_{c,0} + mT_0 \pm \alpha(F_0 + F' mT_0) \tag{1}$$

$$S^{\pm}_m(t^{\pm}_{i,m},\ t^{\pm}_{c,m}) = S_0 \pm \beta F_l(t = mT_0) = S_0 \pm \beta(F_0 + F' mT_0) \tag{2}$$

here the linear-dependence (of t_c and S on the THz field strength) parameters α, β depend on the harmonic order, and are positive/negative for short/long trajectories, respectively. We then separately calculate harmonics generated from the sum of all up or down trajectories,

$$\begin{aligned} j^{\pm}_{\text{inter}}(\omega^{\pm}) &\approx \sum_m \omega^{\pm} F(t^{\pm}_{i,m}) g(t^{\pm}_{c,m}) e^{-(t^{\pm}_{c,m}-t^{\pm}_{i,m})/T_2} e^{i\omega^{\pm} t^{\pm}_{c,m} - iS^{\pm}_m} \\ &\propto \sum_{m=0}^{N-1} e^{i\omega^{\pm}\left(t^{\pm}_{c,0}+mT_0\pm\alpha(F_0+F' mT_0)\right)-i\left(S_0\pm\beta(F_0+F' mT_0)\right)} \\ &= e^{i\omega^{\pm}(t^{\pm}_{c,0}\pm\alpha F_0)-id(S_0\mp\beta F_0)} \sum_{m=0}^{N-1} e^{imT_0(\omega^{\pm}\pm\omega^{\pm}\alpha F' \mp\beta F')} \end{aligned} \tag{3}$$

and,

$$\left|j^{\pm}_{\text{inter}}(\omega^{\pm})\right|^2 \propto \left[\frac{\sin\left(N(\omega^{\pm} \pm \omega^{\pm}\alpha F' \mp \beta F')T_0/2\right)}{N \sin\left((\omega^{\pm} \pm \omega^{\pm}\alpha F' \mp \beta F')T_0/2\right)}\right]^2 \tag{4}$$

the qth "harmonic" frequency $\omega_q^{\pm}$ must satisfy,

$$(\omega_q^{\pm} \pm \omega_q^{\pm} \alpha F^{'} \mp \beta F^{'}) = q\omega_0 \tag{5}$$

therefore,

$$\omega_q^{\pm} = \frac{qw_0 \pm \beta F^{'}}{1 \pm \alpha F^{'}} = qw_0 \pm F^{'} \frac{\beta - \alpha q \omega_0}{1 \pm \alpha F^{'}} \overset{\alpha F^{'} \ll 1}{\simeq} qw_0 \pm F^{'}(\beta - \alpha q \omega_0) \tag{6}$$

For a *cw*-MIR drive, ω_q^{+} and ω_q^{-} are well separated, meaning that the harmonic frequency will be double-spitted with $\Delta\omega_q = \omega_q^{+} - \omega_q^{-} \propto F^{'}$ upon the application of the linearly ramped perturbing field. For a pulsed MIR drive, the harmonic frequency has certain bandwidth, and a quantitative semiclassical analysis becomes challenging. However, the interference between the up and down trajectories, which depends on both F_0 and $F^{'}$, affects the final emitted harmonic frequency.

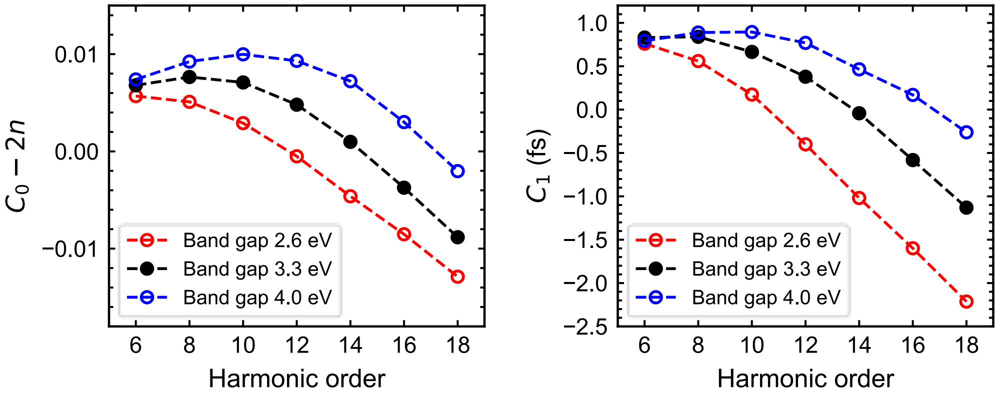

FIG. 15: Harmonic frequency offset ($C_0 - 2n$) and delay-dependent shift (C_1) at three crystal band gap values: 2.6, 3.3, 4.0 eV.

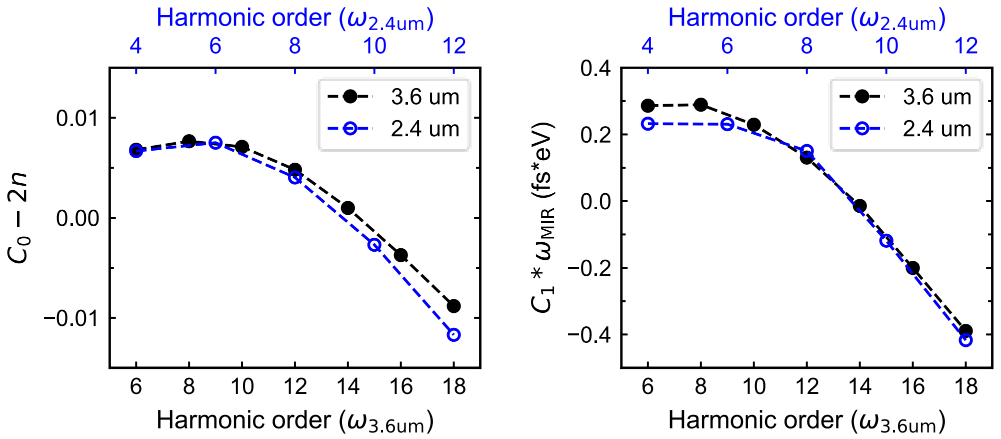

FIG. 16: $C_0 - 2n$ and C_1 at $\lambda_{\mathrm{MIR}} = 2.4$ and 3.6 μm, respectively.

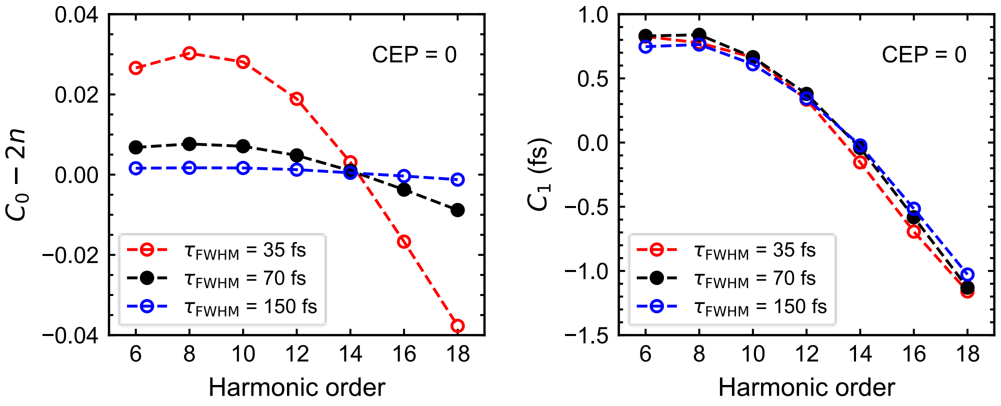

FIG. 17: $C_0 - 2n$ and C_1 at three (intensity FWHM) MIR pulse durations: 35, 70, 150 fs. Simulations with CEP=0 have been shown, however, CEP effects are not significant at these pulse durations.

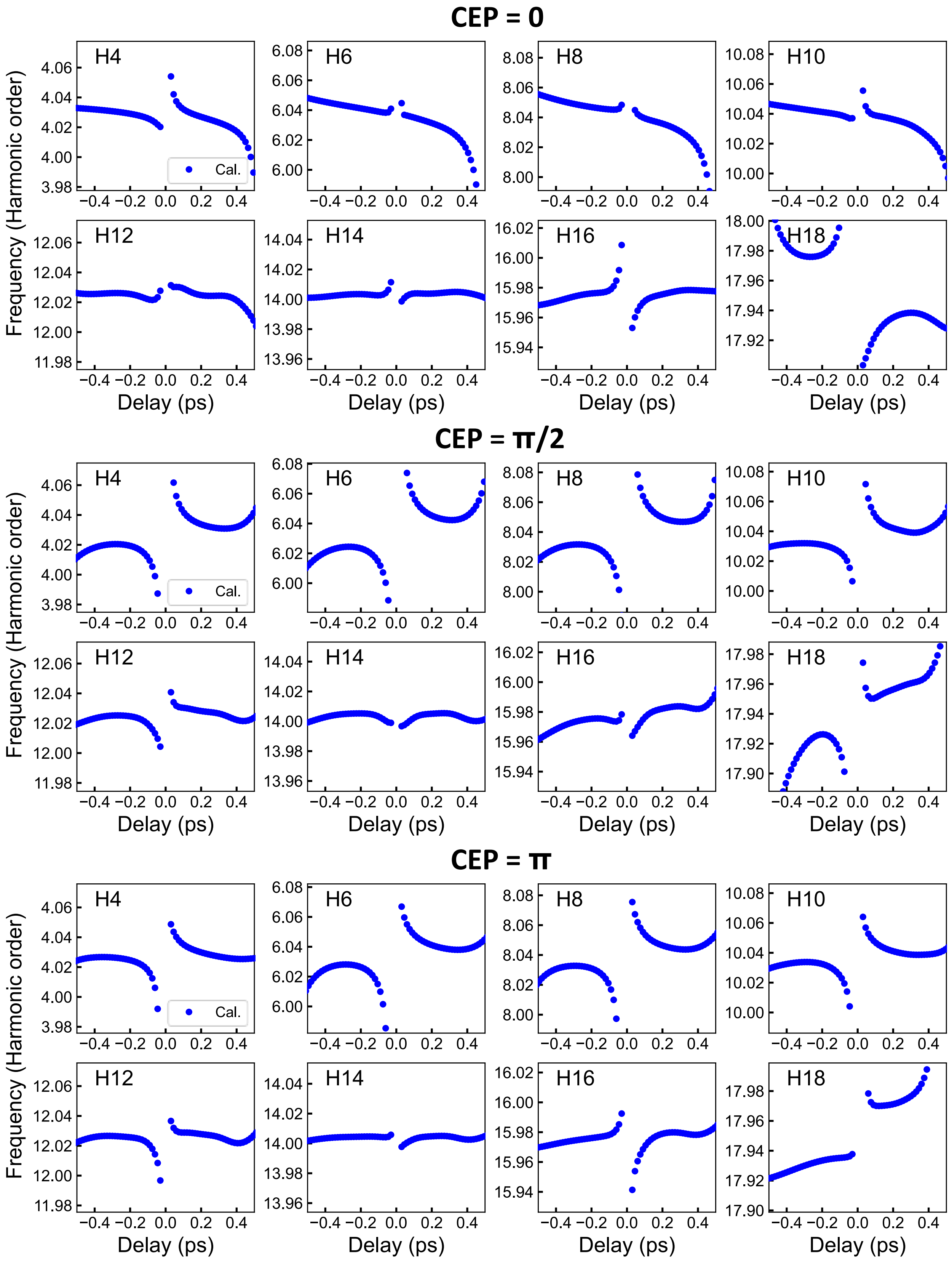

FIG. 18: CEP-dependent even-order harmonic frequency shift with a very short MIR pulse of $\tau_{FWHM} = 30$ fs.

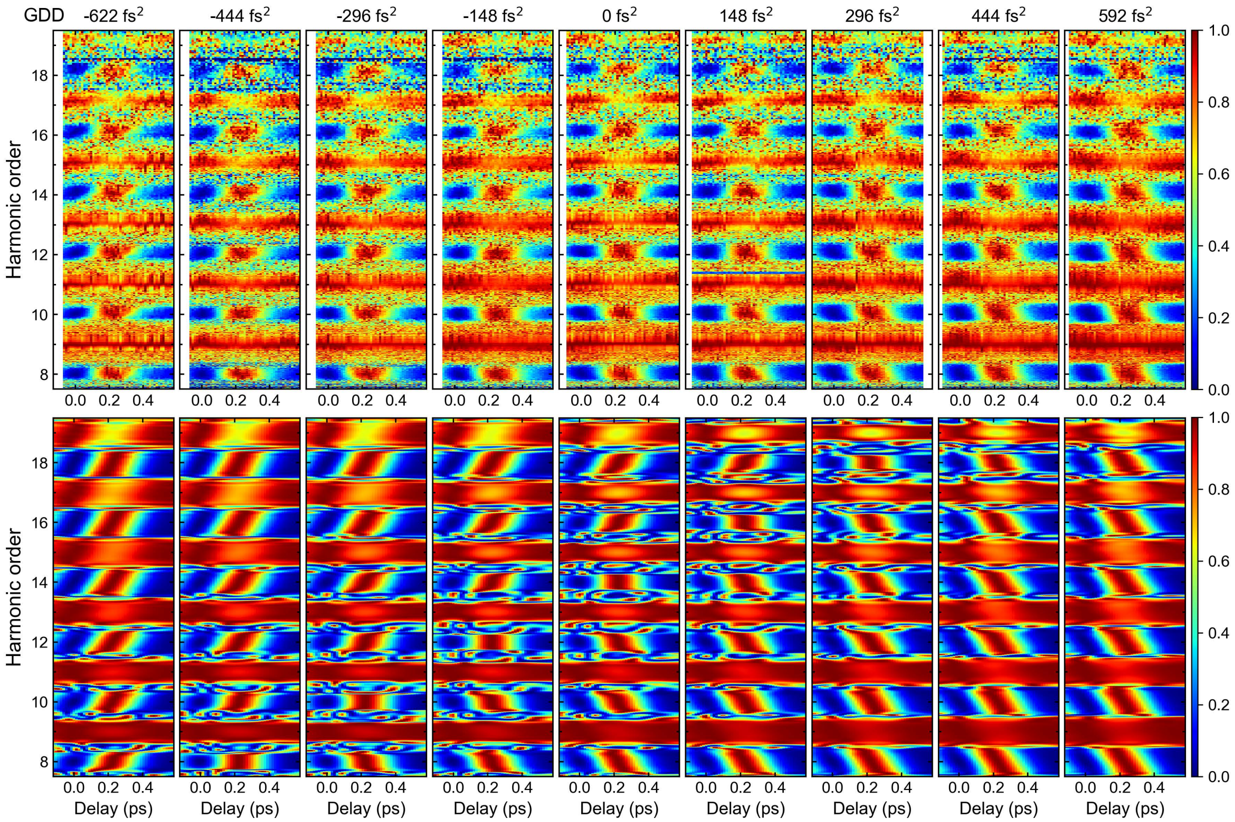

FIG. 19: Even-order harmonic frequency shift at various MIR pulse GDDs. Experiment (top panel), SBEs interband simulation (bottom panel).

---

[1] Huang, H. Terahertz-induced ultrafast symmetry control in condensed matter. (Thesis, Universität Hamburg, 2020).

[2] Shafir, D., Mairesse, Y., Villeneuve, D. M., Corkum, P. B. & Dudovich, N. Atomic wavefunctions probed through strong-field light–matter interaction. *Nat. Phys.* **5,** 412–416 (2009).

[3] Goano, M., Bertazzi, F., Penna, M. & Bellotti, E. Electronic structure of wurtzite ZnO: Nonlocal pseudopotential and ab initio calculations. *J. Appl. Phys.* **102,** 083709 (2007).

[4] Li, B., Zhang, J., Zhang, Y., Yan, T. & Jiang, Y. H. Terahertz-field-induced near-cutoff even-order harmonics in a femtosecond laser. *Phys. Rev. A* **102,** 063102 (2020).
